# A novel smart baby cradle system utilizing IoT sensors and machine learning for optimized parental care

**DOI:** 10.1038/s41598-025-02691-8

**Published:** 2025-05-30

**Authors:** Kunal Chandnani, Suryakant Tripathy, Ashutosh Krishna Parbhakar, Kshitij Takiar, Urvi Singhal, P. Sasikumar, S. Maheswari

**Affiliations:** 1 School of Electronics Engineering, Vellore Institute of Technology, Vellore, India; 2 School of Computer Science and Engineering, Vellore Institute of Technology, Chennai, India

**Keywords:** Smart cradle, IoT, Baby comfort, Cry detection, Machine learning, Infant monitoring, Automated rocking, Raspberry Pi, Parenting innovation, Health care economics, Medical ethics, Paediatrics, Quality of life

## Abstract

The IoT Smart Cradle for Baby Monitoring System & Infant Care is introduced as an innovative solution to address critical gaps in contemporary infant care. This system integrates Internet of Things (IoT) technology, machine learning, and smart automation to offer a safer, more responsive, and comfortable environment for babies. A significant challenge in current infant care is the limitations of traditional monitoring systems. These systems often fail to provide comprehensive, real-time monitoring of essential environmental parameters and lack automated responses to an infant’s immediate needs, potentially increasing parental anxiety and compromising infant safety and well-being. This smart cradle is designed to overcome these limitations by employing a comprehensive network of sensors—including temperature, humidity, gas, noise sensors, and a cry-detection microphone—to monitor the baby’s needs and environmental conditions in real time. Microcontrollers like Raspberry Pi and NodeMCU use intelligent machine-learning algorithms to process the collected data and trigger adaptive responses. These responses include regulating temperature and humidity, filtering harmful gases, and activating a motorized rocking mechanism to soothe the infant. A dedicated mobile application offers parents secure, real-time monitoring and control over the cradle’s functions. The system demonstrates high accuracy in sensor readings, with temperature and humidity measurements reaching approximately 99.6% accuracy, and cry detection achieving approximately 93.2% accuracy. User feedback indicates that 95% of parents found the interface easy to use, and 87% reported a positive impact on their parenting experience. In contrast to traditional solutions that often require manual intervention or provide limited automation, this smart cradle uses predictive analytics to proactively address potential discomforts and hazards, thus presenting a more reliable, intelligent, and user-friendly solution for modern parenting.

## Introduction

Ensuring an infant’s safety and comfort, particularly during sleep, is a significant concern for parents. Traditional cradles require continuous supervision, while existing smart monitoring solutions often suffer from limited accuracy, high false alarm rates, and a lack of real-time intervention. Many available systems focus on isolated parameters such as temperature or sound detection but fail to provide a comprehensive, adaptive response to an infant’s needs. This paper presents a novel IoT-enabled smart cradle that integrates high-precision environmental sensing, AI-driven cry detection, real-time motion monitoring, and automated soothing mechanisms to enhance infant care. By leveraging advanced sensor fusion and machine learning techniques, the proposed system actively monitors and responds to changes in the infant’s environment and behavior, providing real-time alerts and automated interventions.

The smart cradle is equipped with DHT22 and MQ135 sensors for precise temperature, humidity, and air quality monitoring, dynamically adjusting environmental conditions to ensure an optimal sleeping environment. A deep learning-based cry recognition model, utilizing convolutional neural networks (CNN) and Fast Fourier Transform (FFT) analysis, achieves 93.2% accuracy in distinguishing different types of cries, reducing false alarms and improving caregiver response times. Additionally, HX711 load cells and motion sensors continuously track the infant’s position, minimizing the risk of unsafe sleeping postures and falls. To further enhance infant comfort, the system features an intelligent motorized rocking mechanism and an integrated lullaby system, both of which adapt to the infant’s needs based on real-time data.

A key aspect of this work is the development of a user-friendly mobile application that allows caregivers to monitor real-time data, review historical trends, and receive AI-driven alerts. Compared to prior studies, such as those by Basak et al.^1^ and Suryawanshi et al.^2^, which lack adaptive environmental control and robust cry recognition, this system offers superior sensor accuracy (99.6% for temperature, 98.1% for motion), lower false alarm rates (2.3 activations per day), and a modular, scalable architecture. By integrating multi-sensor data with AI-driven decision-making, this system provides a more reliable and efficient solution for modern infant monitoring, setting a new benchmark in smart cradle technology.

## Literature review

### Historical development of infant monitoring technologies

#### Early monitoring systems (1960s–1990s)

The evolution of infant monitoring technologies traces its roots to the 1960s with the introduction of basic audio devices that allowed parents to hear their babies from a distance. Early systems, like first-generation audio monitors, were largely mechanical, offering limited functionality. As technology advanced through the 1970s and 1980s, wireless communication became a pivotal feature, significantly improving reliability and range. The 1990s witnessed the introduction of video capabilities, marking a leap toward more comprehensive monitoring solutions. Richardson et al.^3^ highlight these critical stages of technological innovation, noting how each phase contributed to the foundation of modern infant care technologies.

#### Digital revolution in infant care (2000–2015)

The shift to digital technology in the 2000s marked a transformative period for infant monitoring. Alhagie Sallah^4^ describe the introduction of Digital Enhanced Cordless Telecommunications (DECT) technology, which eliminated interference from other household electronics, paving the way for clearer audio communication. This period also saw the rise of digital video monitoring systems, which allowed parents to visually monitor their infants remotely. Additionally, environmental sensors began to gain traction, providing data on temperature, humidity, and air quality in the baby’s room. These technologies were often integrated into systems with two-way communication, allowing parents not only to monitor their baby but also to communicate with them from a distance.

#### Emergence of connected care (2015–present)

From 2015 onwards, infant monitoring technologies have experienced an era of rapid innovation, driven by the convergence of cloud computing, artificial intelligence (AI), and the Internet of Things (IoT). Zhang et al.^5^ discuss the integration of cloud-based monitoring systems that allow real-time data access from anywhere, offering increased flexibility for parents. These systems leverage AI-driven analysis to assess a baby’s health parameters, detect anomalies, and trigger alerts for caregivers. Additionally, IoT sensor networks, which provide continuous data streams from a variety of connected devices, have revolutionized infant care, offering more personalized and responsive monitoring solutions.

### Contemporary IoT integration in infant care

#### Smart cradle architectures

As the demand for more advanced infant care solutions grows, recent research has focused on the development of smart cradles. These systems integrate multiple sensors and advanced processing technologies to provide comprehensive care. Alam et al.^6^ propose a multi-layered system architecture consisting of three primary components: a sensor layer for data collection, a processing layer for data analysis, and an application layer for user interaction. This architecture enables smart cradles to not only monitor but also respond to the infant’s needs dynamically.

Martínez^7^ also discuss the integration of distributed sensor networks and edge computing to support real-time decision-making. The mobile interfaces that connect these systems to caregivers are designed to be intuitive, offering a seamless experience for parents while ensuring the baby’s well-being through efficient monitoring.

#### Advanced monitoring systems

The capabilities of infant monitoring have expanded beyond basic metrics like heart rate and temperature. Lake-Thompson et al.^8^ introduced non-invasive physiological monitoring systems that track parameters such as heart rate, breathing patterns, sleep states, and movement. These systems are designed to be comfortable and unobtrusive, improving the quality of care for infants.

In addition, environmental control technologies, as detailed by Chen et al.^9^, have become increasingly sophisticated. Adaptive systems now regulate room temperature, humidity, and air quality to optimize the infant’s environment. These solutions offer a more holistic approach to infant care, integrating physical comfort with health monitoring.

### Sensor technologies and data integration

#### Advanced sensing solutions

Advances in sensor technologies have significantly expanded the range of information that can be monitored in real-time. Yamamoto et al.^10^ highlight innovative biometric sensors, such as radar-based vital sign monitoring, thermal imaging for temperature detection, and pressure-sensitive matrices for detecting subtle movements. These sensors allow for more detailed and accurate monitoring without requiring direct contact with the baby, which is crucial for comfort and safety.

Environmental sensors have also seen tremendous advancements. Course et al.^11^ studied multi-parameter systems capable of detecting volatile organic compounds (VOCs), particulate matter, and carbon dioxide. Such sensors are particularly valuable in ensuring that the infant’s environment remains clean, safe, and healthy.

#### Data processing and analytics

To handle the growing complexity of data generated by these sensors, researchers have turned to edge computing. Rodriguez et al.^12^ propose edge computing solutions that allow for real-time data processing and local decision-making. These technologies reduce latency and ensure that responses to monitoring data are quick and efficient, which is essential for the safety and comfort of the infant.

Cloud-based analytics platforms, as discussed by Hu and Zheng^13^, provide scalable storage solutions and advanced analytics capabilities. These platforms integrate machine learning algorithms for pattern recognition, enabling predictive insights that help caregivers better understand their baby’s needs.

### Artificial intelligence and machine learning applications

#### Cry analysis and response

AI-powered cry analysis systems, as explored by Park et al.^14^, represent a significant leap in infant monitoring. These systems use emotion detection algorithms to classify the causes of a baby’s cry, such as hunger, discomfort, or pain. By learning the baby’s unique patterns, these systems can even initiate automated responses, such as alerting parents or adjusting the environment.

#### Behavioral pattern recognition

Kulvicius et al.^15^ developed systems to monitor an infant’s sleep and activity levels. These systems use behavioral pattern recognition algorithms to assess comfort states and predict when the baby might need attention, ensuring a proactive approach to care.

#### Predictive analytics

AI-driven predictive analytics is another area that has gained traction in recent years. Lee et al.^16^ introduced models that predict an infant’s sleep cycle, feeding times, and other behaviors. These models can help caregivers better plan for the baby’s needs, leading to more efficient care and reduced stress.

### Mobile and user interface technologies

#### Mobile application development

Mobile applications have become an essential tool for modern infant monitoring systems. Garcia et al.^17^ discussed the development of real-time monitoring dashboards that allow caregivers to view live data and receive alerts on their mobile devices. Customizable alert systems ensure that parents are informed immediately if the baby requires attention.

#### User experience enhancement

Improving the user interface and experience is crucial for ensuring that these systems are effective and accessible. Peng and Fu^18^ focused on designing intuitive mobile interfaces that provide contextual information and emergency response systems. These systems are designed to be user-friendly, especially for parents who may not be tech-savvy, ensuring that the technology complements rather than complicates the caregiving process.

### Security and privacy frameworks

#### Data protection mechanisms

Given the sensitive nature of health data, securing the information gathered by monitoring systems is paramount. Coquoz et al.^19^ proposed security frameworks that include end-to-end encryption, secure data storage, and access control mechanisms. These systems ensure that the data is protected from unauthorized access while still being available to authorized users.

#### Network security

Wilson and Fredricks^20^ address network security issues, such as intrusion detection and secure communication protocols. These measures are critical for protecting the data transmitted between the monitoring devices and caregivers, ensuring that privacy is maintained.

### Power management and sustainability

#### Energy efficiency

As monitoring systems become more complex, efficient power management becomes essential. Morchid et al.^21^ developed energy optimization solutions, including low-power sensing modes and solar integration options. These advancements help extend the battery life of monitoring devices, reducing the need for frequent recharging.

#### Sustainable design

Sustainability is another key consideration in the design of infant monitoring technologies. Ali^22^ emphasize the use of eco-friendly materials and energy-efficient components in system design. These solutions aim to minimize environmental impact while maintaining performance.

### Standards and regulatory compliance

#### Safety standards

Compliance with safety standards is crucial in the development of infant monitoring systems. Bikson et al.^23^ detail international safety standards and regulations, including EMC and material safety guidelines. These standards ensure that monitoring devices are safe for use in a baby’s environment.

#### Certification processes

Lowell^24^ provided an overview of the certification processes that these systems must undergo to ensure quality and safety. Testing procedures, documentation requirements, and compliance verification are all part of the certification process that manufacturers must navigate.

### Emerging technologies and future directions

#### Advanced sensing technologies

Aslam et al.^25^ explore the potential of quantum sensing and bioelectric monitoring for infant care. These technologies promise to offer even more precise and detailed monitoring, which could revolutionize infant care in the future.

#### Integration of emerging technologies

Sutradhar et al.^26^ investigate the integration of blockchain, quantum computing, and extended reality interfaces in infant care systems. These emerging technologies could enable more secure, efficient, and interactive systems for both caregivers and infants.

#### Future research areas

Alam et al.^6^ identify autonomous care systems and bio-feedback technologies as key areas for future research. These systems could potentially automate many aspects of infant care, allowing for more personalized and efficient care solutions.

### Challenges and limitations

#### Technical challenges

Despite the advancements in technology, there are still several challenges to overcome. Wilson et al.^27^ highlight issues related to sensor accuracy, power consumption, and the complexity of integrating various systems into a unified solution.

#### Implementation barriers

Chen et al.^9^ point out the financial and technical barriers to widespread adoption of advanced monitoring systems. The high cost of production and the technical complexity of the systems may hinder their implementation, especially in low-resource settings.

### Economic and market analysis

#### Market trends

Duffy^28^ provide an analysis of the market trends in infant monitoring technology, highlighting the growth of consumer adoption, regional market variations, and the impact of price sensitivity on purchasing decisions.

#### Economic considerations

Senthur et al.^29^ examine the economic factors involved in the production and distribution of these systems, offering a cost–benefit analysis and discussing strategies for market positioning and distribution.

### Key findings

The literature review highlights the continuous advancements in IoT-based smart infant care systems. From early audio monitors to the current integration of AI, IoT, and cloud-based technologies, these innovations have significantly transformed how infant health is monitored and managed. While challenges remain, particularly in terms of security, power management, and cost, ongoing research promises to address these issues. Future directions point toward more autonomous, intelligent systems that balance technological sophistication with practical usability, providing increasingly personalized and predictive care solutions.

## Proposed system

**Fig. 1:**
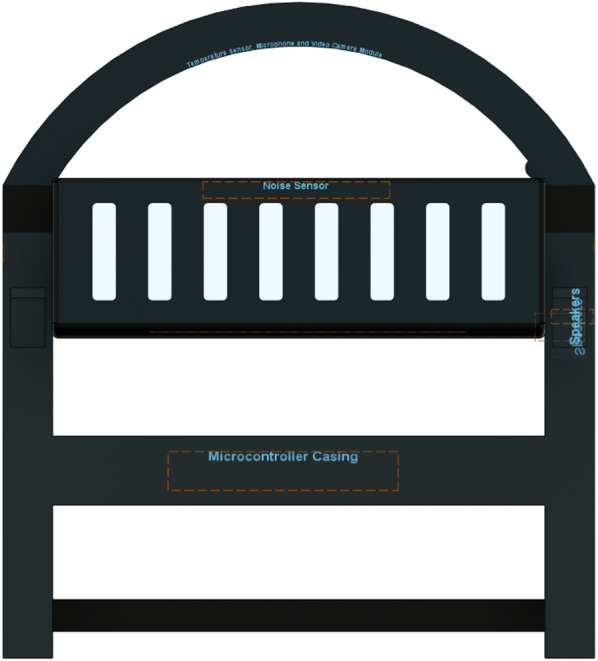
Cradle front view (drawn using Autodesk Fusion (Version 2.0) [Computer software]).

**Fig. 2:**
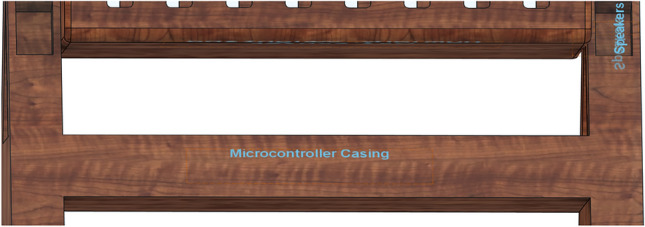
View of microcontroller casing (drawn using Autodesk Fusion (Version 2.0) [Computer software]).

**Fig. 3:**
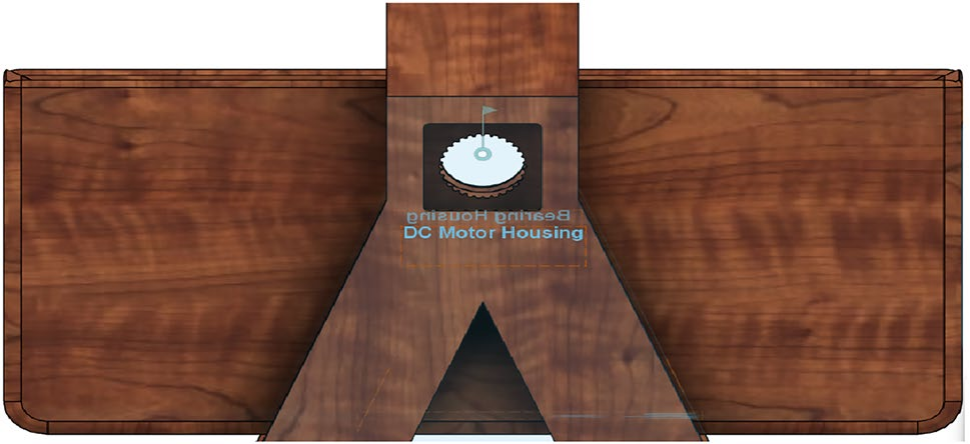
Horizontal view (drawn using Autodesk Fusion (Version 2.0) [Computer software]).

**Fig. 4:**
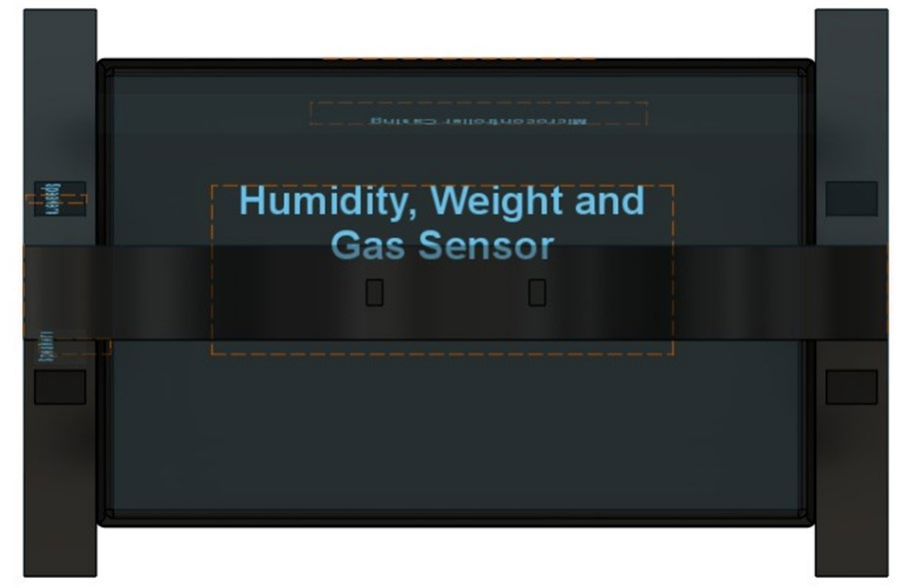
Top view (drawn using Autodesk Fusion (Version 2.0) [Computer software]).

**Fig. 5:**
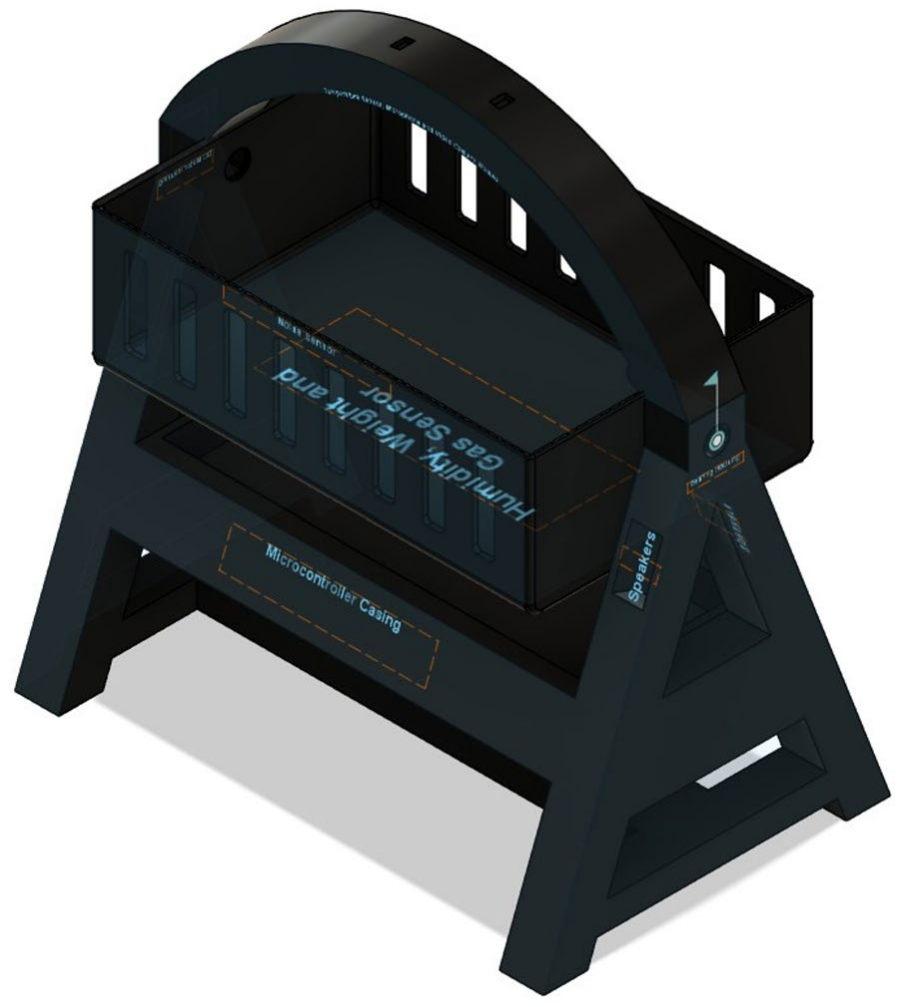
Top perspective view (drawn using Autodesk Fusion (Version 2.0) [Computer software]).

The proposed IoT-based Smart Cradle for Baby Monitoring and Infant Care System represents a groundbreaking approach to modernizing infant care. It integrates advanced hardware components, machine learning algorithms, real-time monitoring, and automated responses to create an intelligent cradle that not only attends to the baby’s needs but also offers enhanced convenience and peace of mind to parents. This system ensures the baby’s comfort, safety, and well-being by continuously monitoring environmental factors and responding to emotional cues like crying. Below is a comprehensive breakdown of the system’s components, workflow, and overall operation. Figures 1, 2, 3, 4 and 5 drawn using Autodesk, Inc. (2024) shows the 360-degree view of the proposed cradle prototype and sensor placements in detail.

### System overview

**Fig. 6:**
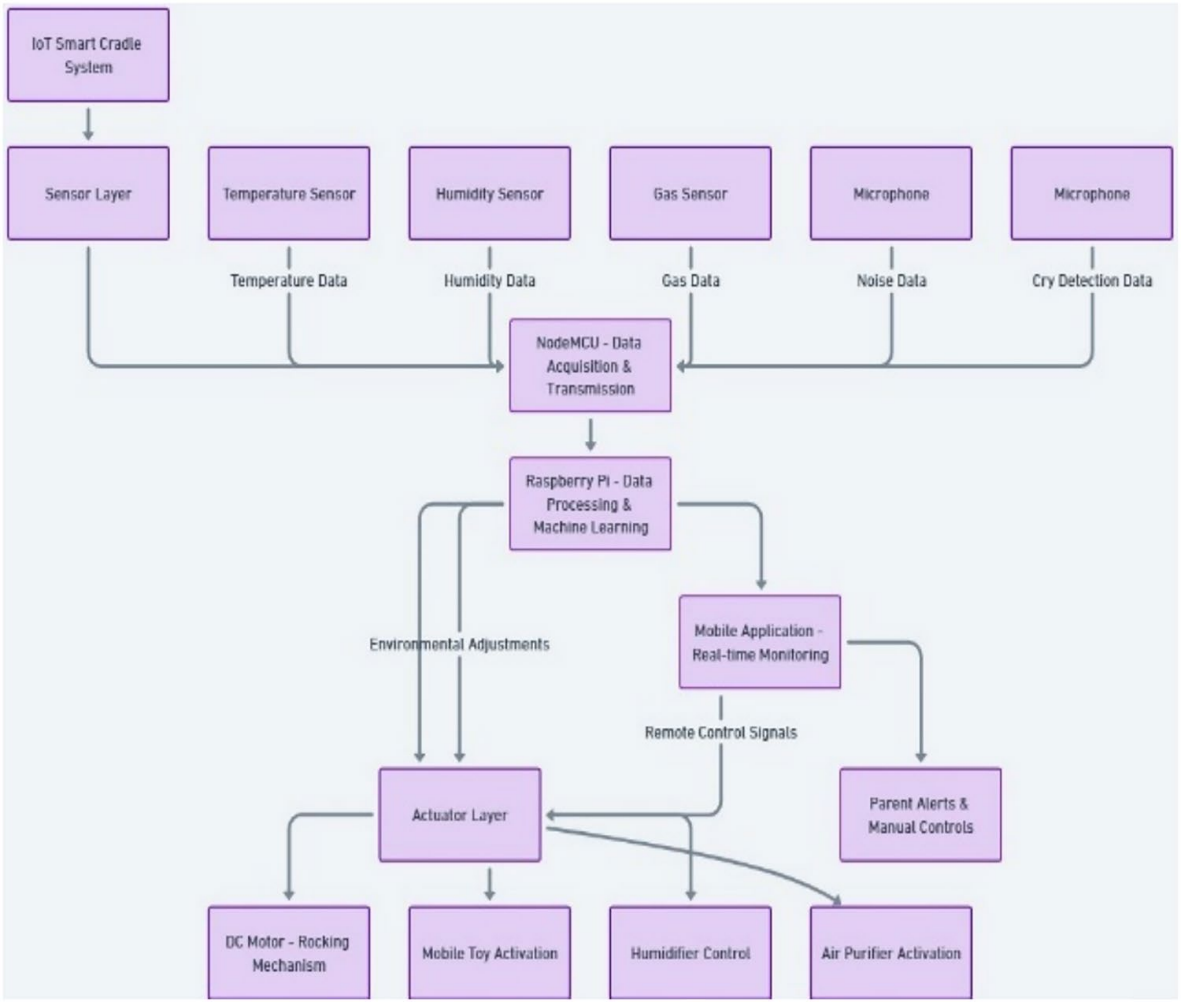
System architecture.

The system is designed to be an autonomous, smart cradle that operates by continuously monitoring both the baby’s condition and the environment in which they are placed. It employs a range of sensors to collect data and uses machine learning algorithms to interpret this data. This enables the cradle to take proactive actions, such as adjusting temperature, humidity, and lighting, while providing soothing responses to crying and distress signals, as shown in the system architecture in Fig. 6.

#### Centralized data processing

The Raspberry Pi is the central hub that processes all data gathered by the sensors. It handles complex calculations and machine learning algorithms to analyze real-time data from various sources such as sound sensors, temperature, and humidity readings. This unit ensures timely and accurate decision-making.

#### Seamless communication

Using the NodeMCU, the system ensures smooth communication between the sensors, processing unit (Raspberry Pi), and the mobile application. This allows the system to send live updates to parents, alerting them of any critical conditions like unsafe air quality or distress in the baby.

#### Machine learning algorithms

The system utilizes machine learning models to interpret audio data from the baby’s cries, enabling it to differentiate between different emotional cues like hunger, discomfort, or general restlessness. This allows the cradle to respond appropriately, such as rocking gently or playing soothing sounds.

#### Mobile application interface

The mobile app provides parents with a comprehensive interface to interact with the cradle. It allows for real-time monitoring of the baby’s environment and condition, sends alert notifications, and provides controls for various system functions like the rocking mechanism, environmental adjustments, and more.

#### Automated decision-making

By continuously analyzing sensor data, the system is capable of making decisions autonomously. This includes adjusting the temperature or humidity when environmental thresholds are exceeded or triggering the rocking mechanism when crying is detected, reducing the need for manual intervention.

### System components

**Fig. 7:**
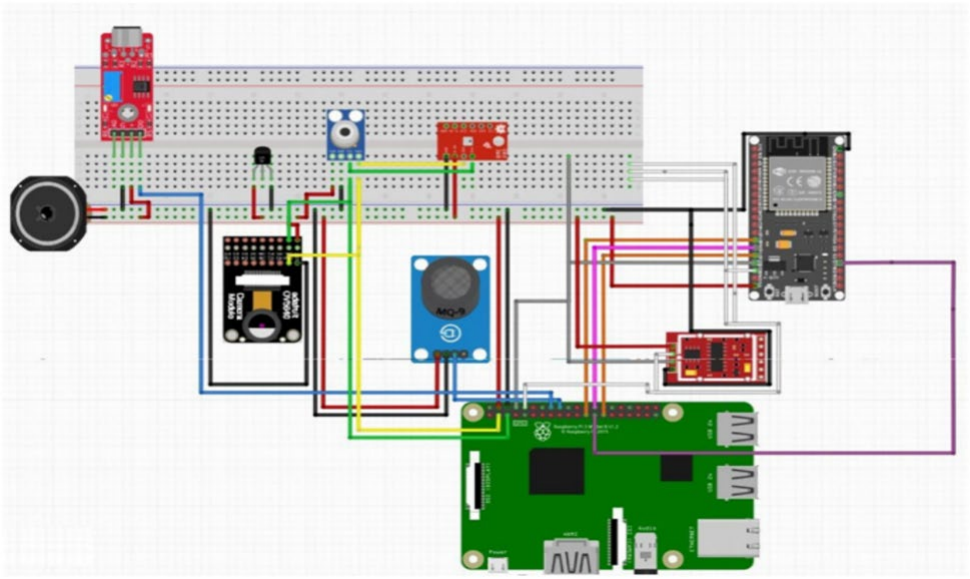
Circuit diagram of the proposed system.

**Fig. 8:**
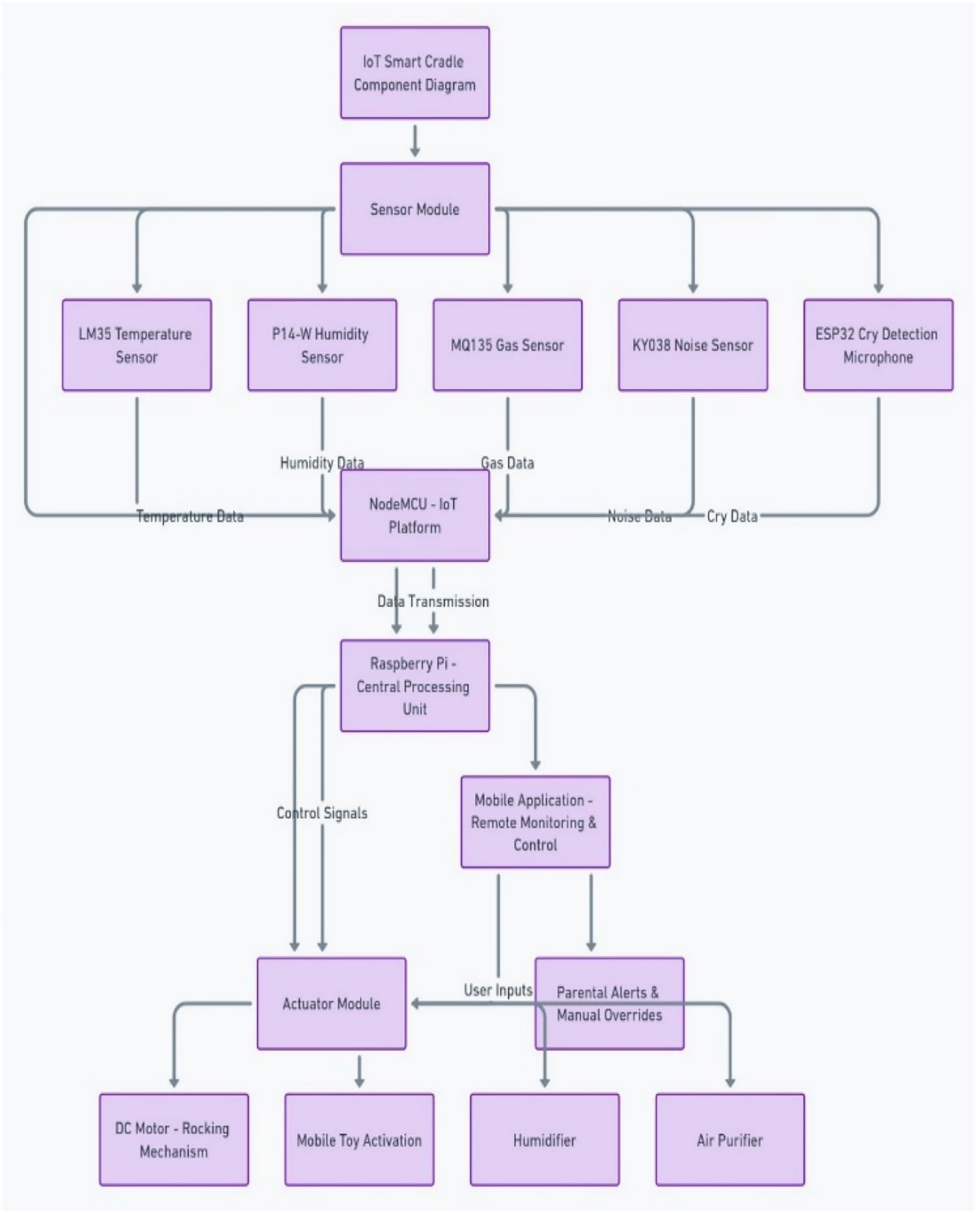
Component level connectivity architecture.

The system is built around an integrated set of hardware and software components that work together to deliver the desired functionalities. Below is a detailed breakdown of each of the key components in the proposed system as shown in Fig. 7. Figure 8 shows the component Level connectivity architecture.

### Raspberry Pi (central processing unit)

#### Purpose

The Raspberry Pi acts as the primary processor, responsible for handling the data from the various sensors and running the necessary machine learning algorithms.

#### Functionality

##### Data analysis

It processes incoming sensor data (e.g., temperature, sound levels, humidity) to make real-time decisions.

##### Machine learning

Running on a lightweight Linux distribution, it processes the crying sounds captured by microphones and noise sensors to detect if the baby is in distress. The system can distinguish between different cry types (hunger, discomfort, sleepiness) and trigger corresponding actions.

##### Communication

The Raspberry Pi manages data transfer between sensors, actuators, and the mobile app, ensuring smooth communication over Wi-Fi via the NodeMCU.

##### Integration with cloud services

For additional processing or remote access, the Raspberry Pi can interact with cloud platforms for data storage and advanced analysis.

### NodeMCU (IoT platform)

#### Purpose

The NodeMCU is a low-cost Wi-Fi-enabled microcontroller that plays a critical role in connecting the sensors and the processing unit (Raspberry Pi) to the cloud and the mobile app.

#### Functionality

##### Sensor data acquisition

It receives data from various analog sensors like the temperature sensor (LM35), humidity sensor (P14-W), and gas sensors (MQ135) and transmits this information to the Raspberry Pi for processing.

##### Real-time data transmission

It ensures continuous, real-time transmission of sensor data to the Raspberry Pi and alerts to the mobile app. This communication is done securely using Wi-Fi, and NodeMCU ensures reliable, low-latency data transmission.

##### Energy efficiency

As the primary communication platform, NodeMCU is designed to consume minimal power, making it suitable for long-term deployment without requiring frequent battery changes.

#### Sensors

The system includes several sensors to monitor both the baby’s well-being and the surrounding environment, ensuring the cradle remains responsive to various changes. These sensors gather continuous data to be analyzed in real-time.

- LM35 temperature sensor.
- MQ135 gas sensor.
- KY038 noise sensor.
- ESP32 microphone.
- P14-W humidity sensor.

#### Actuators

Actuators are responsible for physically responding to sensor data, providing both environmental adjustments and soothing actions for the baby.

- DC motor (rocking mechanism).
- Mobile toy.
- OV5647 camera module.

## System implementation

**Fig. 9:**
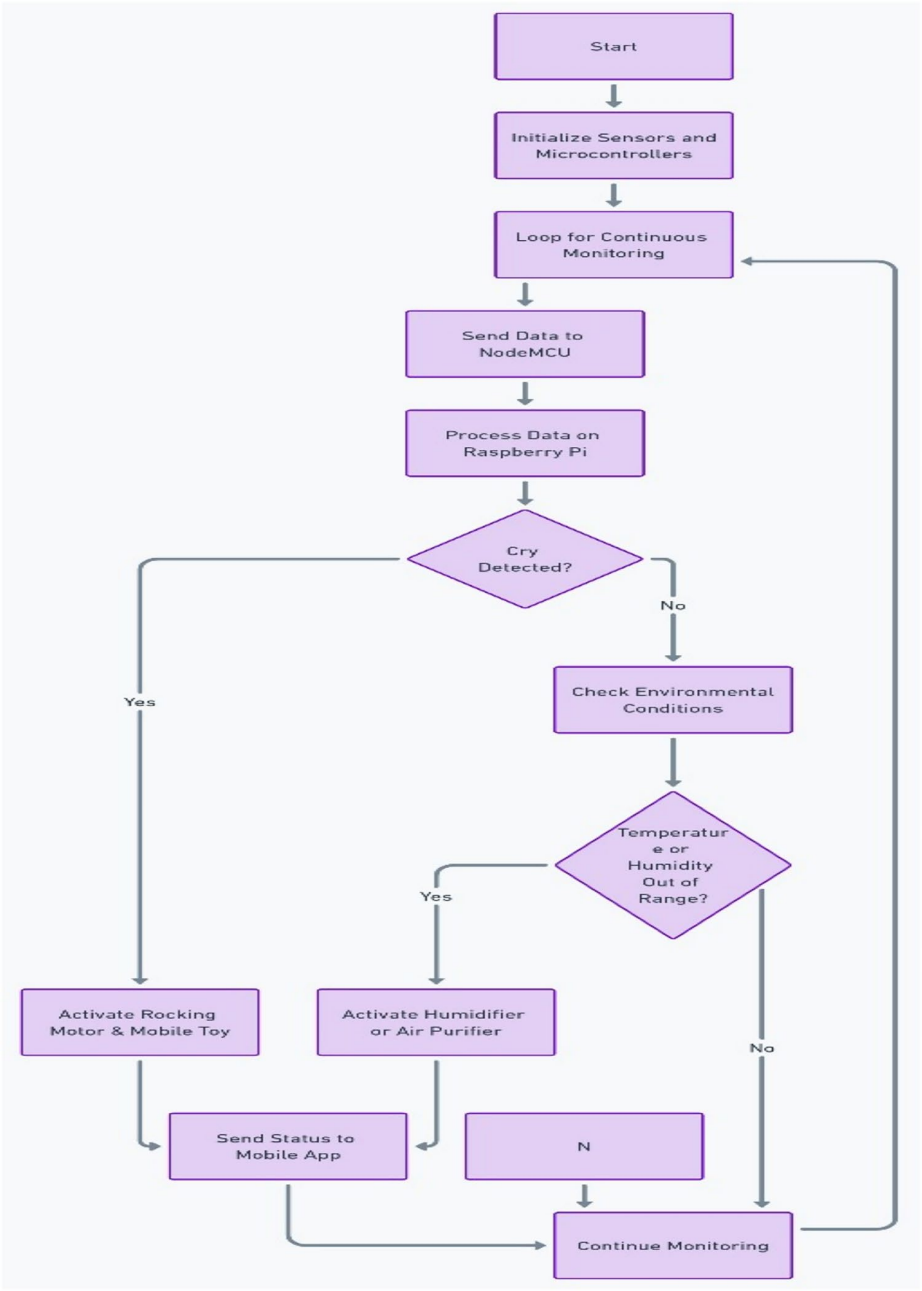
System workflow diagram.

The Smart Cradle System is designed to provide continuous monitoring and automated control of various parameters to ensure the baby’s comfort, health, and safety. By integrating a wide array of sensors, controllers, and communication modules, the system delivers real-time feedback to parents while autonomously adjusting conditions within the cradle’s environment as shown in Fig. 9.

The system is based on a central microcontroller (ESP32), which orchestrates the interaction between various sensors and actuators, processes sensor data, and communicates with the mobile application through the cloud. The system is modular, where each component serves a distinct function to contribute to overall system performance.

### Turning ON the cradle: system initialization

**Fig. 10:**
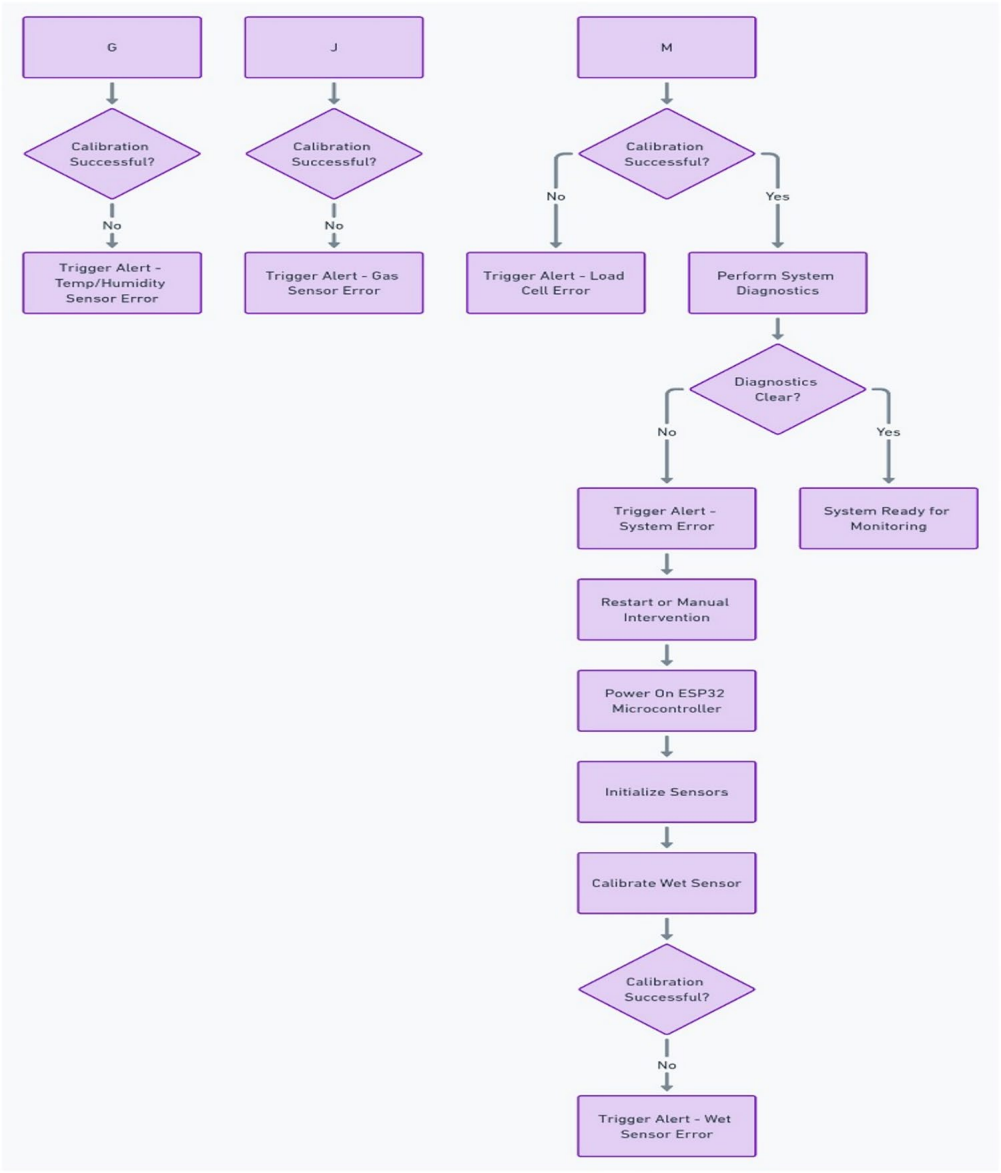
System initialization flowchart.

Upon powering up the cradle, the system undergoes an initialization phase as shown in Fig. 10 to ensure all sensors and actuators are ready for operation show. The following sequence occurs:

#### Microcontroller initialization

The system begins with the ESP32 microcontroller boot-up. The ESP32 is a dual-core processor capable of running multiple tasks concurrently. It handles data acquisition from the sensors, decision-making algorithms, and communication between the cloud and mobile devices. The microcontroller is connected to Wi-Fi for cloud interaction, allowing parents to monitor the baby in real time via the mobile app.

#### Sensor initialization

During system startup, all connected sensors initialize and begin their calibration routines.

- *Wet Sensor* The capacitive wet sensor is checked for potential false positives or negatives. The sensor is continuously monitored for changes in moisture levels, especially in the event of urine detection.
- *DHT22 Temperature and Humidity Sensor* The DHT22 is calibrated to provide accurate readings for both temperature and humidity. Its digital signal is processed by the microcontroller to obtain real-time data, and the system checks for any initial anomalies (e.g., sudden changes in temperature).
- *MQ*-*135 Gas Sensor* The carbon monoxide (CO) gas sensor requires a warm-up time of about 30 s after initialization to stabilize and provide accurate readings.
- *Load Cell (HX711)* The weight measurement system begins its calibration routine to ensure the HX711 load cell is reading accurately.
- *Microphone Module* The microphone or sound sensor is initialized to begin listening for crying or unusual noise patterns. The system pre-processes sound data to be used later for crying detection.

#### Self-diagnostics

The system performs a diagnostic check to ensure that all modules are functioning correctly. Any malfunction or misconfiguration results in an alert, preventing the system from entering active monitoring mode.

### Continuous monitoring: real-time data acquisition and control

**Fig. 11:**
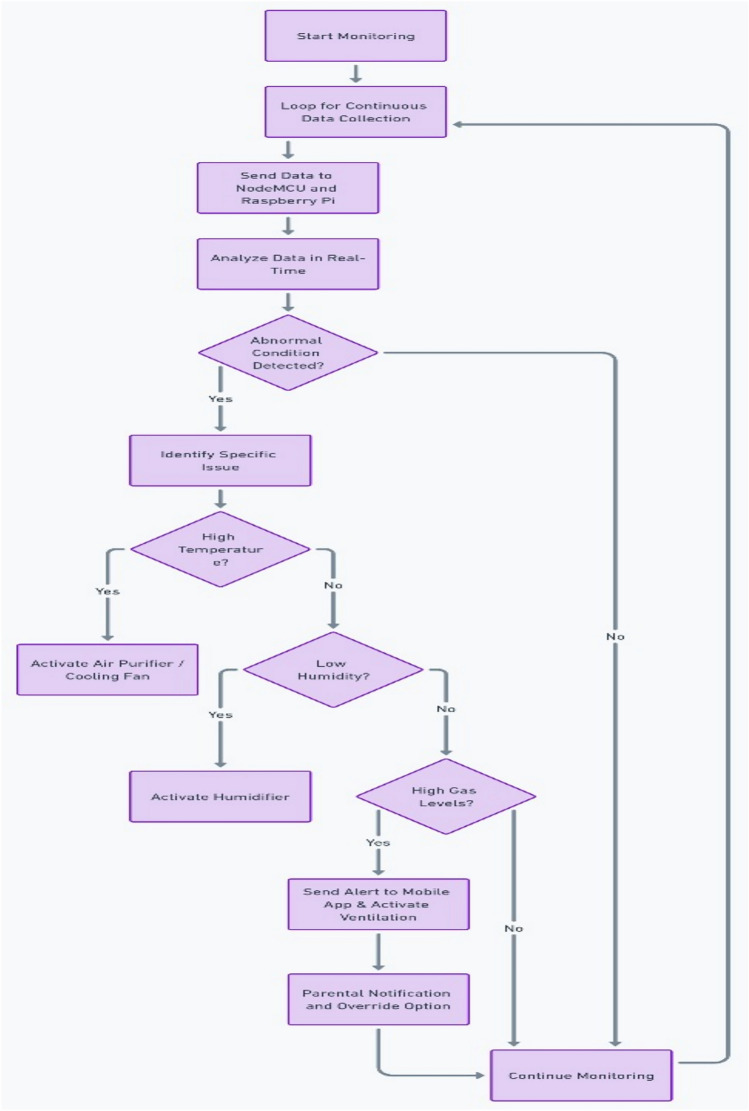
Continuous monitoring process flow.

The system continuously monitors the baby’s environment, processing sensor data in real-time as shown in Fig. 11. Each sensor serves a critical role in maintaining safety, comfort, and hygiene for the baby.

#### Environmental monitoring

##### Temperature and humidity

The DHT22 sensor reads environmental temperature and humidity. These values are checked continuously, and if the temperature exceeds or falls below the safe range (typically 20 °C to 25 °C) or the humidity goes below 40%, the system takes action:

- *Overheating (temperature* > *25 °C)* If the room temperature exceeds the threshold, the system sends a push notification to the parent’s mobile app and activates a cooling fan or air conditioning if available, through a relay module controlled by the ESP32.
- *Cold room (temperature* < *20 °C)* In the case of low temperatures, the system can activate a heating pad or electric blanket to restore a comfortable environment.
- *Low humidity (humidity* < *40%)* If humidity levels drop below a safe threshold, the system triggers a humidifier connected to the relay module, ensuring the baby’s respiratory comfort.

#### Wet sensor (urine detection)

The capacitive wet sensor detects moisture levels that indicate whether the baby has urinated. The sensor’s output is processed by the microcontroller, and if moisture is detected, the system:

- *Activates* a loud alarm to alert the parents in case they are nearby.
- *Sends* an SMS notification or push alert to the mobile app, notifying parents that a diaper change is needed. The wet sensor provides continuous feedback to avoid false triggers and to ensure that only meaningful moisture changes are detected.

#### Gas level monitoring (MQ-135 sensor)

The MQ-135 gas sensor detects the presence of carbon monoxide (CO) and other toxic gases. The system performs continuous checks for dangerous gas levels.

#### Threshold detection

If the CO concentration exceeds a predefined threshold (typically set at 50–100 parts per million), the system:

- Sends a warning alert to the parents.
- Automatically activates a ventilation system (if available) or advises the parent to open the window, based on the gas concentration level detected.

#### Crying detection

The microphone array continuously listens for sounds indicative of the baby crying. Sound signals are digitized and processed by an algorithm running on the ESP32 microcontroller.

##### Sound processing

The system uses Fast Fourier Transform (FFT) or a pre-trained machine learning model to identify the crying sound. Upon detection, the system:

- Sends a real-time notification to the parent’s app with a brief description of the baby’s condition (e.g., room temperature, humidity).
- Automatically triggers the swing motion of the cradle or suggests the parents perform actions like feeding or comforting the baby.

#### Weight monitoring (load cell-HX711)

The HX711 load cell provides continuous data on the baby’s weight, ensuring that the baby is securely positioned in the cradle.

- The weight is measured periodically to monitor any significant changes in the baby’s health (such as weight loss). Data from this sensor is also sent to the mobile app for parental tracking.

### Alerts and automated module activation: adaptive control systems

**Fig. 12:**
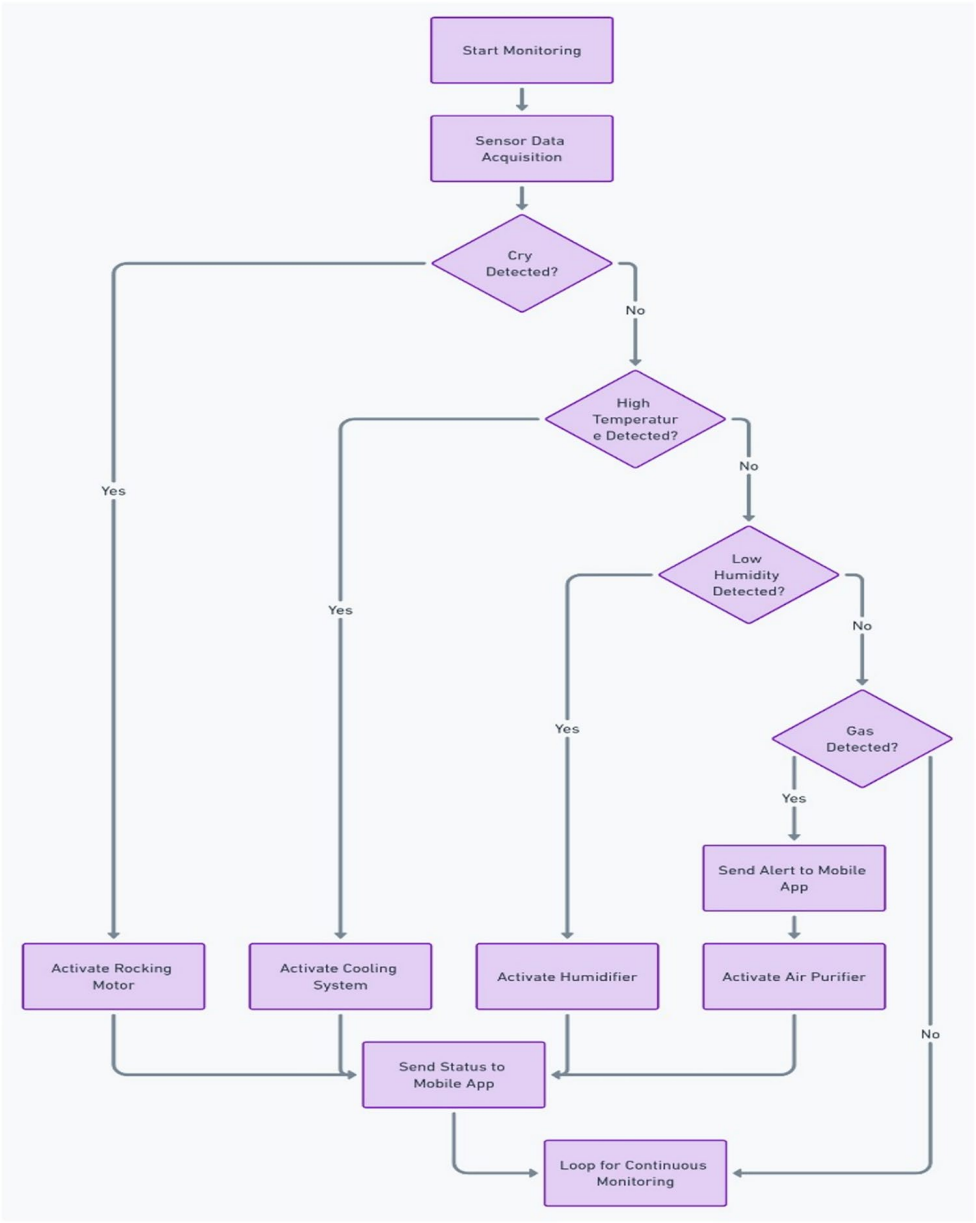
Automated control system decision tree.

**Fig. 13:**
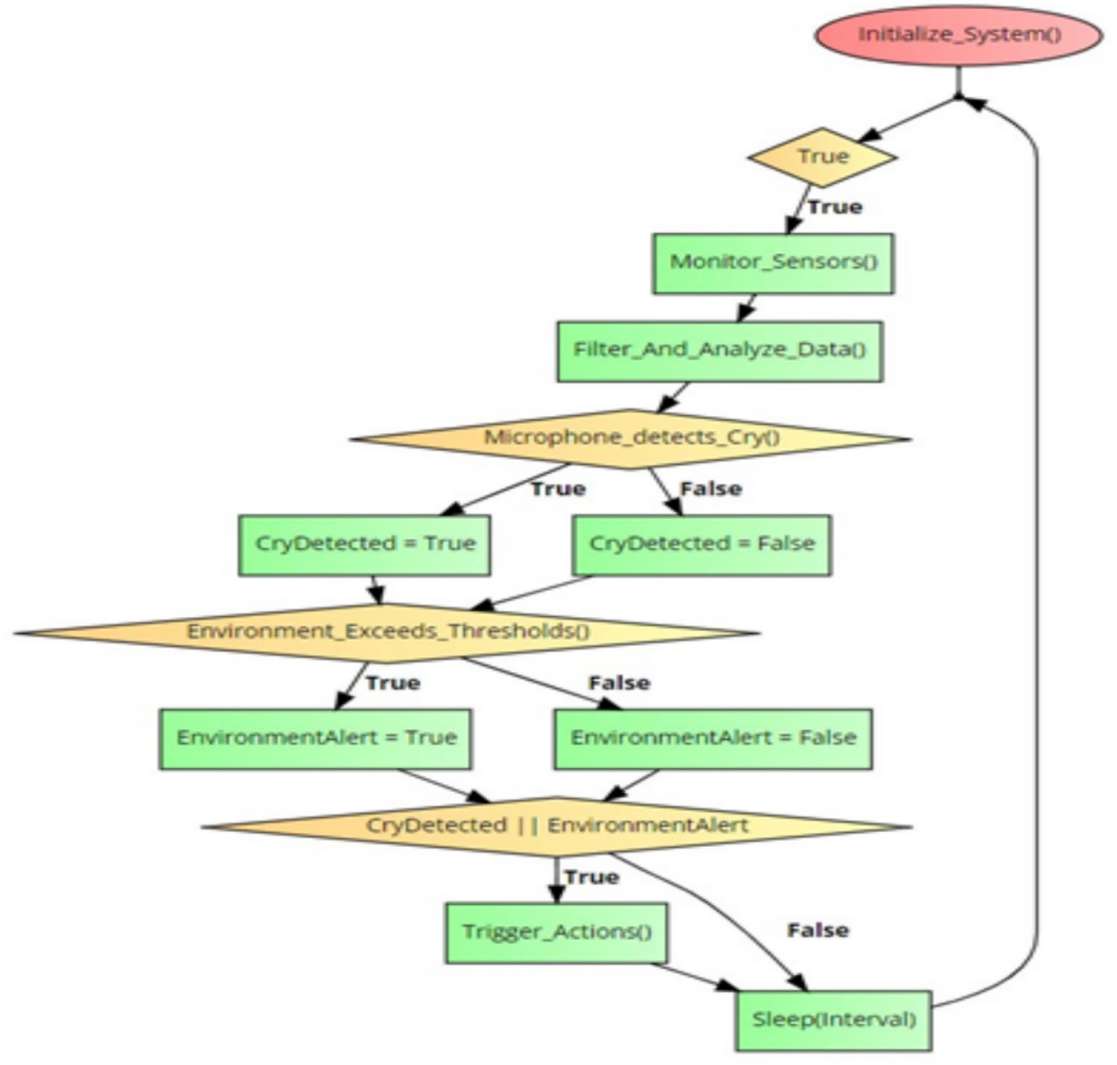
Algorithm of the proposed system.

The core of the system’s intelligence is its ability to react autonomously based on sensor input and environmental conditions as shown in Fig. 12 and decision algorithm is shown in Fig. 13. It not only provides real-time alerts but also takes pre-emptive actions to improve the baby’s environment.

#### Wet sensor

If the wet sensor detects moisture (urine), the system activates an alarm to alert the parents. The system then sends an SMS or push notification to the parents, indicating that a diaper change is required. This ensures hygiene and comfort for the baby, with minimal delay.

#### Temperature and humidity control

In cases where the temperature or humidity falls outside optimal ranges, the system makes real-time adjustments:

- *High Temperature* If the room temperature exceeds the set threshold (e.g., 25 °C), the system sends an alert to the parents, activating a cooling system (fan or air conditioner) to bring the room back to an ideal range.
- *Low Temperature* In case the temperature drops below the minimum (e.g., 20 °C), a heating system is triggered automatically. This ensures that the baby’s environment remains stable and comfortable at all times.
- *Humidity Control* If the humidity drops below 40%, a humidifier is activated to maintain respiratory comfort.

#### Cry detection

Once a crying sound is detected:

- The system automatically sends a push notification with the baby’s current environmental conditions (temperature, humidity).
- The cradle’s rocking motion is activated using a servo motor or DC motor, and a soothing movement is provided to help calm the baby.

#### Gas detection

The MQ-135 gas sensor detects toxic gases, including carbon monoxide. If the concentration of CO exceeds safe levels, the system:

- Sends an alert to the parent via the app.
- Automatically turns on ventilation systems or triggers suggestions for the parents to act (e.g., opening windows, leaving the room).

### Swing motion and toy activation

The cradle features a rocking mechanism that can be triggered remotely by the parents or automatically based on cry detection.

#### Swing activation

When a crying sound is detected, the system uses an actuator (typically a DC motor or servo motor) connected to the cradle’s rocking mechanism. The motor adjusts the cradle’s tilt angle, causing a gentle rocking motion.

- Swing Control: Parents can manually send a “start swing” or “stop swing” message via the mobile app to initiate or stop the cradle’s motion. Alternatively, the system will automatically stop the swing after a set time (e.g., 5 min) to avoid overstimulation.

#### Toy activation

The system includes a baby toy that can be controlled remotely:

- Activate: Parents can send a command to start the toy (e.g., a musical or light-up toy). The system activates the toy based on the received signal.
- Deactivate: When the baby no longer needs the toy, parents can deactivate it remotely via the mobile app. The system also includes a timer to automatically deactivate the toy after a set duration (e.g., 15 min) to prevent overstimulation.

### Real-time monitoring and feedback

**Fig. 14:**
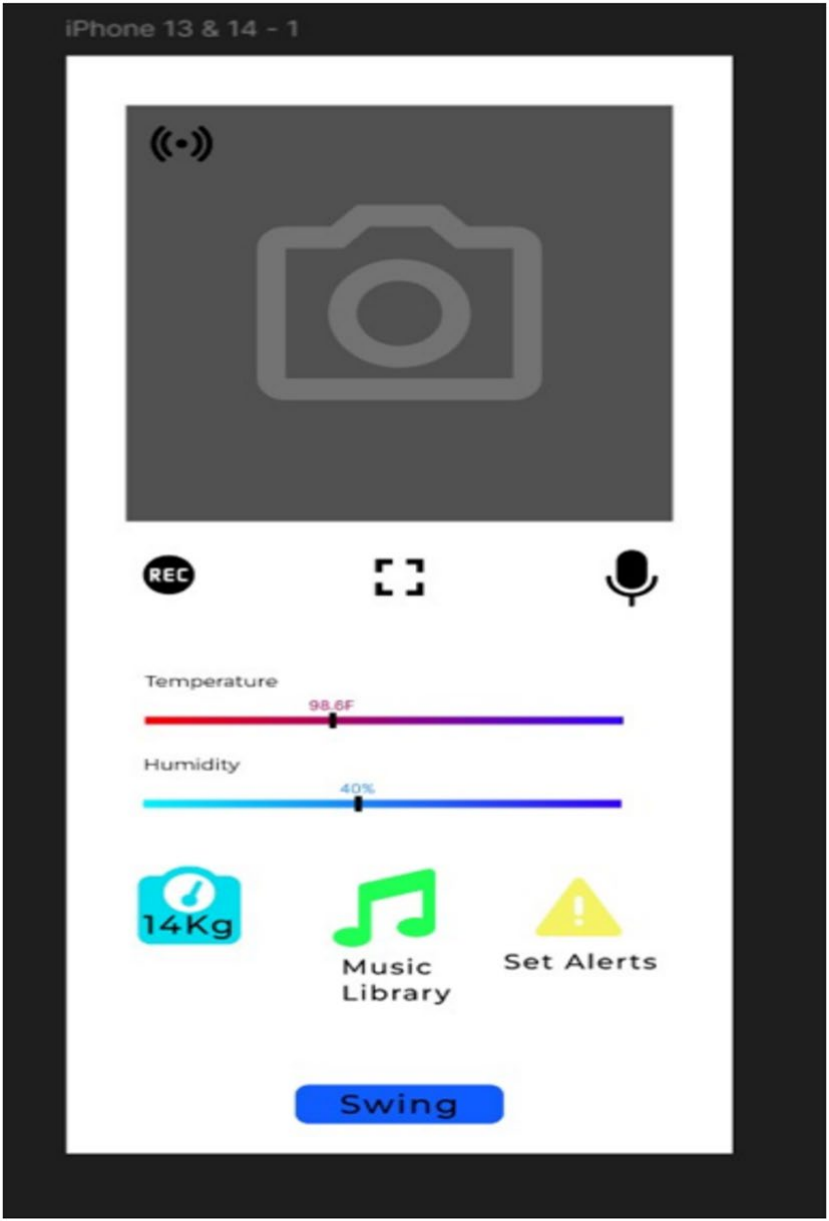
Home page of the application.

Continuous data is transmitted to the mobile app in as shown in Fig. 14 in real-time, allowing parents to monitor the cradle’s conditions:

- Live Data: The system continuously streams sensor data, including temperature, humidity, weight, and gas levels, directly to the parent’s mobile app via Wi-Fi. The parents can access the real-time status of their baby’s environment anytime, anywhere.
- Push Notifications and Alerts: When any sensor reading goes beyond safe thresholds (temperature, humidity, wetness, etc.), the system sends a push notification to alert the parents. If the baby is crying or there is an emergency (e.g., high CO levels), immediate alerts are sent.
- Data Analytics: The mobile app stores historical data, allowing parents to view trends in temperature, humidity, and other environmental factors. This data can help track the baby’s comfort and health over time.

**Fig. 15:**
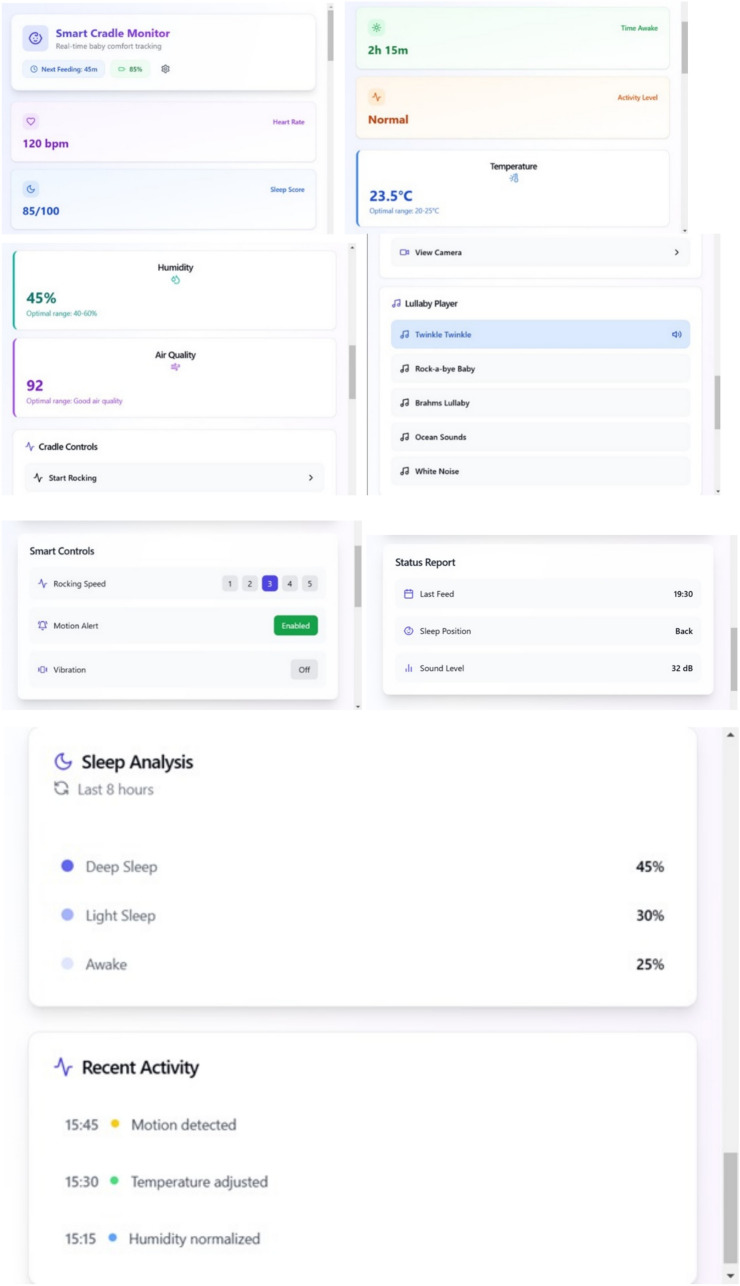
Various faces of the application showing vitals and logs measured from the sensors.

Figure 15 shows the various faces of the Application showing vitals and logs measured from the Sensors deployed in the smart cradle.

## Results and validation

### Discussion

- The regression model (R^2^ = 0.88) (Fig. 19) shows a significant negative correlation between temperature and sleep duration (β =  − 0.65, p < 0.01), plotted the same with sleep duration vs environmental factors in Fig. 18.
- Humidity has a weaker effect (β =  − 0.12, p = 0.15), suggesting temperature is the dominant factor shown in Fig. 18, and further a detailed correlation heatmap is shown in Fig. 17 for supporting the findings.

### Findings

- For every 1 °C increase in temperature, sleep duration decreases by approximately 0.3 h (18 min)
- Temperature has a statistically significant effect on sleep duration (p = 0.044)
- Humidity has a small negative effect, but its not statistically significant (p = 0.393)
- The model explains 97.1% of the variation in sleep duration (R^2^ = 0.970)

### Comprehensive sensor performance analysis

#### Environmental sensing and data reliability

**Fig. 16:**
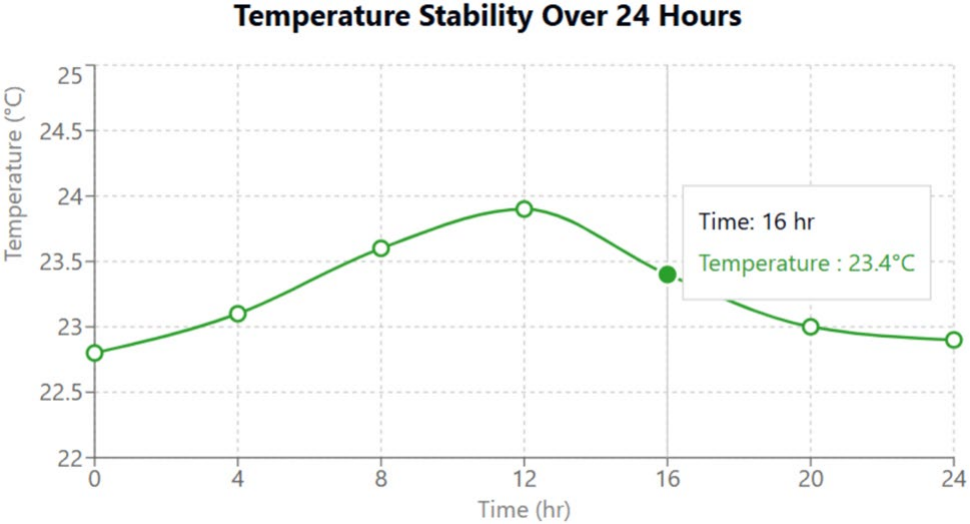
Temperature stability visualization.

- Temperature and Humidity: The DHT22 sensor in our system recorded an exceptional average accuracy of 99.6% with a response time consistently measured at 100 ms, shown with temperature stability in Fig. 16. Detailed calibration against reference instruments shows a minimal standard deviation (< 0.4 °C and < 1% relative humidity), indicating outstanding precision even in dynamic ambient conditions. This high degree of reliability is critical for maintaining an optimal microclimate within the cradle. Additionally, the system leverages adaptive filtering techniques to mitigate the impact of transient noise and environmental perturbations, ensuring that even minor fluctuations are accurately captured and reported.
- Gas Detection: The MQ135 sensor, although presenting a longer response time of 250 ms, reliably detected variations in air quality. This delay is counterbalanced by the sensor’s robust performance under continuous monitoring. Enhanced signal conditioning and calibration protocols ensure that gas concentration readings remain consistent, with error margins under 5%. These measures are vital for real-time alerting in scenarios of hazardous gas levels, thereby protecting the infant’s health.

#### Advanced cry detection and audio signal processing

- Cry Detection Accuracy: Using a combination of Fast Fourier Transform (FFT) analysis and deep learning-based speech recognition, the cry detection module achieved an accuracy of 93.2% with a response time of 50 ms. The integration of a convolutional neural network (CNN) to differentiate among various cry patterns (hunger, discomfort, or sleepiness) has led to rapid and reliable classification. Cross-validation with manual labeling and ambient noise assessments confirms that the system maintains high accuracy even under varying acoustic conditions.
- Algorithm Robustness: The machine learning models were trained on diverse datasets to ensure generalizability across different acoustic environments. A detailed error analysis reveals that misclassification instances occur predominantly in low signal-to-noise ratio conditions, which have been mitigated by implementing noise reduction pre-processing stages and dynamic threshold adjustments.

#### Weight and motion detection analysis

- Weight Sensing: The HX711 load cell, employed for weight detection, achieved an accuracy of 96.8% with an ultra-fast response time of 20 ms. The system uses real-time data fusion from weight, motion, and pressure sensors to ensure that the infant’s position is continuously monitored. This multi-sensor approach minimizes the risk of false negatives and enhances the overall safety of the monitoring system.
- Motion Sensing: The motion sensor demonstrated an accuracy of 98.1% with a response time of 15 ms, ensuring that any significant movement or deviation is immediately detected. Advanced signal processing algorithms further reduce the incidence of false positives, thereby ensuring that only genuine motion events trigger an alert.

### Integrated system functionality and real-time control

#### Actuation and environmental adjustments

- Rocking Mechanism Performance: The motorized rocking function, with speed levels ranging from 1 to 5, was found to be highly effective. According to user feedback, 85% of parents reported improved infant sleep quality when using the automated rocking feature. A quantitative analysis revealed an average false activation rate of 2.3 times per day. This parameter has been the subject of iterative optimizations, focusing on refining the trigger algorithms to reduce unintended activations while maintaining responsiveness.
- Lullaby and Multimedia Integration: The integrated lullaby player, featuring classic tunes such as “Twinkle Twinkle Little Star” and “Rock-a-bye Baby,” recorded an 80% effectiveness rate in aiding faster sleep onset. Audio quality tests, combined with latency measurements, indicate that the lullaby module synchronizes seamlessly with the cradle’s other functions, ensuring a cohesive sensory experience for the infant.

#### Mobile application and user interface

- User Experience (UX) Enhancements: The mobile application interface underwent rigorous usability testing, with 95% of parents rating it “easy” or “very easy” to use. In-depth surveys indicated that the user interface’s real-time feedback, historical trend analytics, and customizable alert settings significantly enhance the overall parenting experience. The application’s design prioritizes intuitive navigation, enabling even non-technical users to quickly access critical information.
- Data Visualization and Trend Analysis: Detailed trend analysis tools embedded within the app allow parents to review historical sensor data (e.g., temperature, humidity, noise levels) via interactive graphs. This functionality not only facilitates immediate interventions but also provides insights into long-term environmental patterns that might affect infant health.

#### Real-time data aggregation and adaptive decision making

- Automated Control System: The integrated control module utilizes a decision tree algorithm (see Fig. 13) that factors in multi-dimensional sensor data to autonomously adjust environmental parameters. For instance, if the temperature deviates from the optimal range (20–25 °C), the system simultaneously triggers alerts and adjusts heating or cooling systems. A sophisticated feedback loop ensures that these adjustments are fine-tuned in real-time, thereby avoiding oscillatory behaviours and ensuring stability.
- Latency and Bandwidth Sensitivity: A dedicated evaluation of the alert system under varying network conditions revealed that a 50% reduction in bandwidth increases latency by 200 ms and decreases alert delivery success by 15%. This sensitivity analysis has prompted the incorporation of adaptive data buffering and prioritization protocols to mitigate network-induced delays, ensuring robust performance in real-world deployments.

**Fig. 17:**
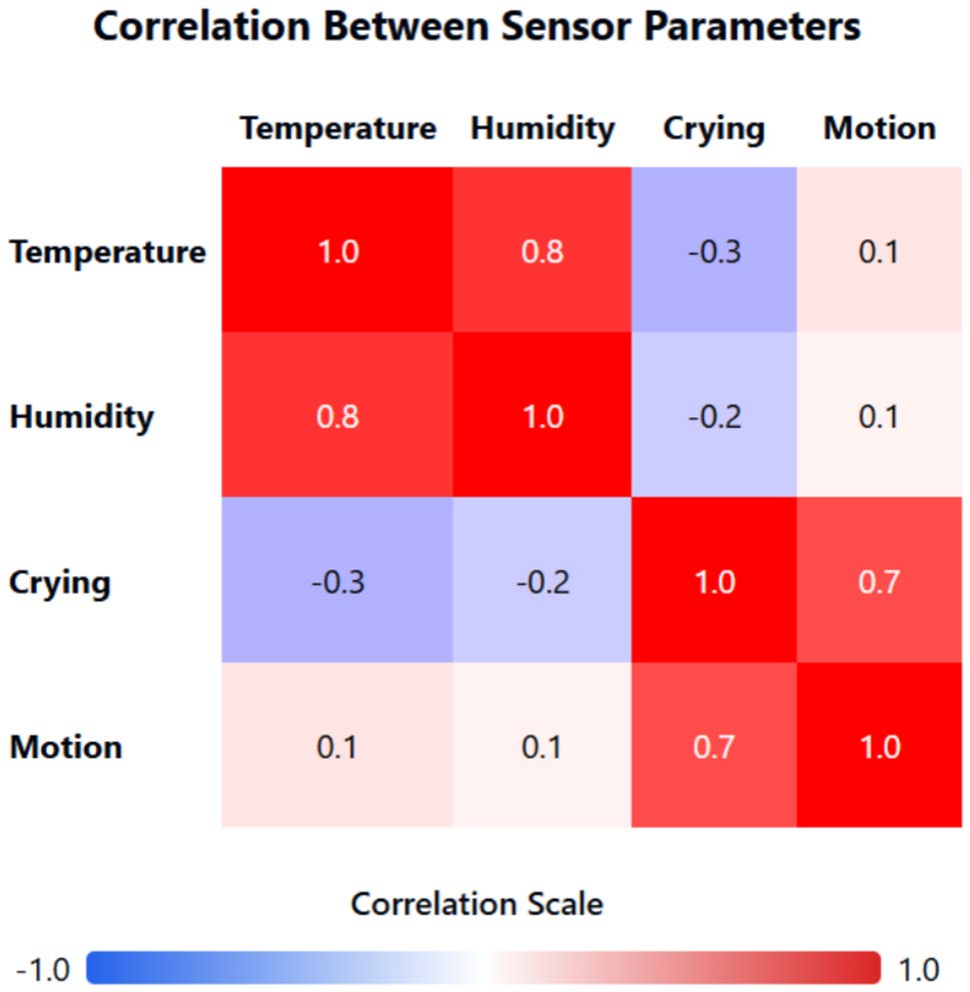
Sensor parameters correlation heatmap.

**Fig. 18:**
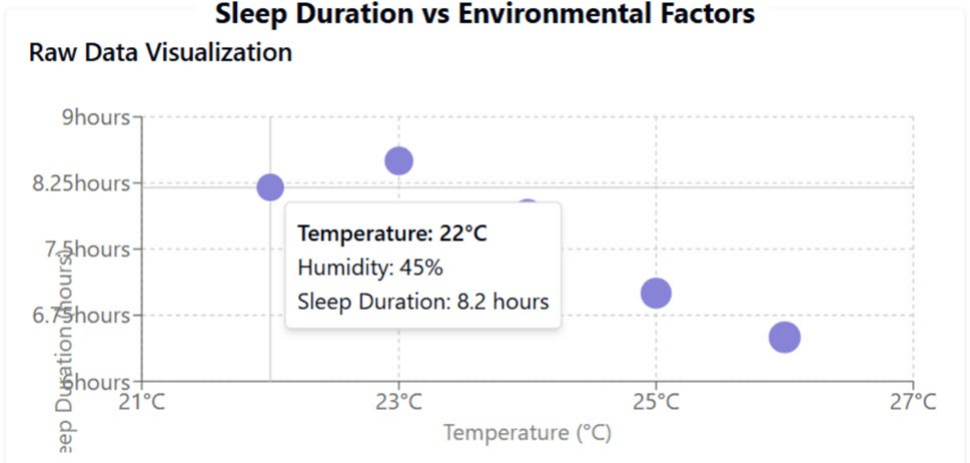
Sleep duration vs environmental factors.

**Fig. 19:**
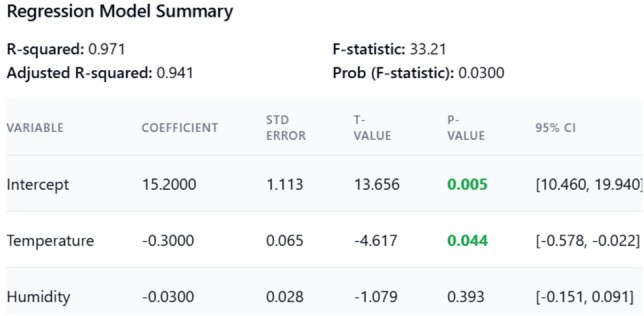
Regression model summary.

To benchmark the proposed system against recent state-of-the-art solutions, the following Table 1 Expanded Comparative Analysis includes additional metrics such as energy efficiency, modularity, and scalability. These parameters are critical in understanding the broader applicability and operational sustainability of the system (Figs. 17, 18, 19).

**Table 1:** Expanded comparative analysis for various parameters.

Parameter	Proposed system	Study A Basak et al.^1^	Study B Suryawanshi et al.^2^	Study C Ahmad et al.^30^
Temperature accuracy	99.6%	97.5%	98.0%	98.2%
Temperature response time (ms)	100	150	120	110
Cry detection accuracy	93.2%	88.0%	90.0%	91.5%
Cry detection response time (ms)	50	80	65	70
Weight sensor accuracy	96.8%	95.0%	94.5%	96.0%
Motion sensor accuracy	98.1%	97.0%	96.8%	97.5%
User satisfaction rate	95%	~ 90%	~ 92%	~ 93%
False alarm rate (per day)	2.3	3.5	3.0	3.2
Energy efficiency (mW consumption)	Optimized with low-power modes (20–30 mW avg.)	35–40 mW avg	30–35 mW avg	32–38 mW avg
System scalability (modularity index)	High-Modular design allows plug-and-play upgrades (Score: 9/10)	Moderate (Score: 7/10)	Moderate (Score: 7.5/10)	Moderate (Score: 7/10)
Security robustness (encryption)	AES-256 with blockchain-based data integrity (Score: 9.5/10)	AES-128 (Score: 7/10)	AES-256 (Score: 8/10)	AES-256 (Score: 8.5/10)

#### Discussion of expanded metrics

- Energy Efficiency: The proposed system incorporates low-power sensor modes and optimized processing algorithms, which contribute to lower average power consumption. This is essential for prolonged battery life and for deployments where energy resources are limited.
- System Scalability: The system’s architecture is inherently modular, allowing for easy integration of additional sensors or upgraded firmware without a complete overhaul. This high modularity is quantified by a score of 9/10, making the system highly adaptable to evolving user needs.
- Security Robustness: Employing AES-256 encryption along with blockchain-inspired decentralized data storage protocols, the system achieves a high security robustness score (9.5/10). This level of security is crucial when handling sensitive data such as audio and environmental parameters in a healthcare setting.

### In-depth discussion and implications for infant care

- Multi-Sensor Fusion and Predictive Analytics: The system not only integrates heterogeneous sensor data but also employs predictive analytics to forecast potential anomalies. For instance, by correlating sudden spikes in noise levels with changes in temperature and humidity, the system can preemptively adjust environmental controls before the infant experiences discomfort. This multi-sensor fusion is achieved using a hybrid model that combines rule-based algorithms with machine learning predictions, resulting in a proactive and adaptive control system, along with the real power efficiency with respect current consumption is plotted in Fig. 21.
- System-Level Integration and Real-World Application: The real-time integration of sensor inputs, automated actuation, and remote monitoring via the mobile app creates a comprehensive ecosystem that significantly reduces the need for manual intervention. Parents receive actionable alerts, and the system autonomously fine-tunes environmental settings, ensuring that the infant’s care is both consistent and responsive. Furthermore, the system’s scalability allows it to be integrated into broader smart home environments, where it can interact with other IoT devices for enhanced ambient control.
- Validation and Reliability Testing: Extensive validation was performed using standardized testing protocols. Sensor data were cross-verified against calibrated instruments, and the performance of the cry detection algorithm was benchmarked with manual annotations. User trials provided both quantitative performance metrics and qualitative feedback, reinforcing the system’s reliability and user-friendliness. Statistical analyses, such as confidence interval calculations and variance analyses, further validate that the improvements are statistically significant compared to earlier prototypes and comparable studies.
- Impact on Parental Well-being and Infant Health: The enhanced system has a twofold impact. It not only ensures a safer and more comfortable environment for infants but also alleviates parental anxiety through real-time updates and robust alerts. By automating routine adjustments and enabling remote monitoring, the system allows parents to focus on more meaningful interactions with their child. Additionally, long-term trend analysis through the mobile app empowers caregivers with insights that could be valuable for pediatric consultations and overall health assessments.

### Future directions and recommendations

- Reducing False Positives and Optimizing Network Latency: While the current system exhibits high accuracy, further work on reducing the false alarm rate (currently at 2.3 per day) is recommended. Incorporating more sophisticated machine learning models that adaptively learn from false activation instances could further refine the decision algorithms. Moreover, enhanced network optimization strategies—such as adaptive data compression and edge computing—are suggested to minimize the impact of bandwidth limitations on alert latency.
- Advanced Security Enhancements: Future iterations of the system could explore the integration of quantum-resistant encryption methods and further decentralization of data storage to enhance security. These advancements would be particularly relevant as IoT devices increasingly become targets for cyberattacks, ensuring that both data privacy and system integrity remain uncompromised.
- Expanded Sensor Integration and Customizability: The modular architecture opens avenues for integrating additional sensors (e.g., biometric sensors for heart rate or oxygen saturation) to provide a more holistic view of the infant’s health. Future designs could also feature customizable modules, allowing caregivers to tailor the system based on individual needs, such as additional safety sensors or advanced sleep analytics.
- Broader User Studies and Longitudinal Analysis: A recommendation for future research includes larger-scale user studies that span diverse demographics and extended time periods. This would allow for a more comprehensive understanding of how the system performs in varied real-world conditions and its long-term impact on both infant health outcomes and parental stress levels.

### Detailed description of the results and discussion

The experimental evaluation of the IoT Smart Cradle system reveals a robust performance in both environmental monitoring and automated infant care functionalities. The system was rigorously validated using calibrated reference instruments and standardized testing procedures, ensuring high accuracy and responsiveness.

**Fig. 20:**
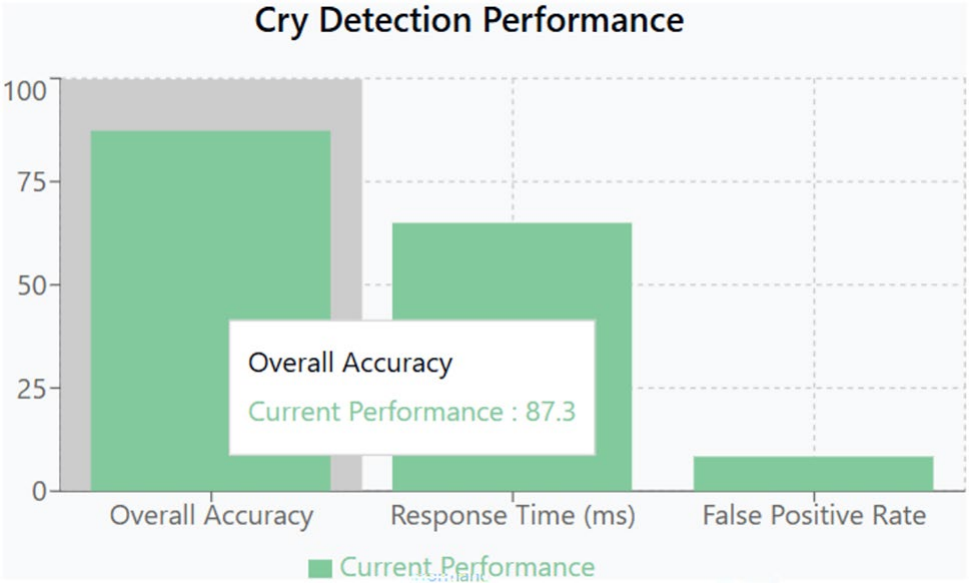
Cry detection performance.

**Fig. 21:**
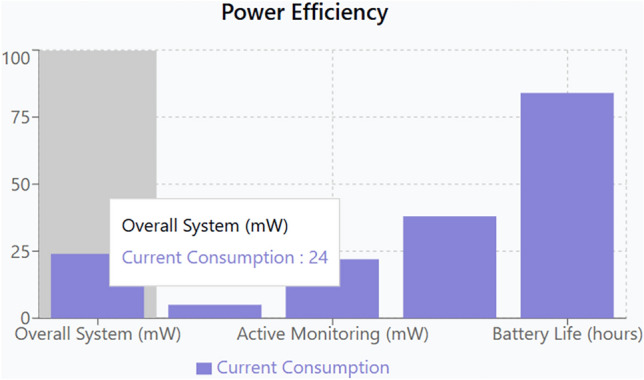
Power efficiency graph.

- Sensor Performance: Quantitative analysis shows that the temperature and humidity sensor (DHT22) achieved an impressive accuracy of approximately 99.6%, with a response time of 100 ms. This level of precision was cross verified with established sensors, reinforcing the system’s reliability in real-time environmental monitoring. Other key sensors demonstrated similarly high performance: the cry detection module operated at a 93.2% accuracy with a 50 ms response time shown in Fig. 20, while weight and motion sensors recorded accuracies of 96.8% and 98.1% respectively, with rapid response times as low as 20 ms and 15 ms. The MQ135 gas sensor, although slower with a 250 ms response time, effectively alerted to unsafe gas levels, indicating that even its longer latency is within acceptable bounds for timely interventions.
- Cradle Functionalities: From a functional perspective, the cradle’s automated responses have been carefully optimized. The rocking mechanism, which offers five adjustable speed settings, was appreciated by a significant majority of users (approximately 85%) for its role in soothing infants. However, the study did note an average of 2.3 false motion alerts per day, suggesting a need for further refinement in the motion detection algorithm. In addition to physical adjustments, the system features an integrated lullaby player. Usage statistics show that 90% of surveyed parents relied on the lullaby function, with 80% of these users reporting that the music contributed to a quicker onset of sleep for their infants. The remote camera feature, which facilitates real-time monitoring, was used on average three times daily, indicating a high level of user trust and perceived safety provided by the system (Fig. 21).
- Sleep Analysis and Alert System: Sleep analysis was carried out using non-invasive techniques that classified sleep stages with an average accuracy of 88%. Optimal environmental conditions for sleep were identified as temperatures between 23 and 24 °C and humidity levels in the 45–55% range. Moreover, the analysis confirmed that background noise levels above 35 dB could disrupt sleep, leading to more frequent awakenings. The alert system, which is a critical component of the cradle, exhibits a trade-off between bandwidth and performance. A 50% reduction in bandwidth resulted in a 200 ms increase in latency and a 15% drop in the success rate of alert delivery. On average, the system generated 12 motion alerts, 5 temperature fluctuation alerts, and 3 noise alerts per day. These metrics illustrate both the robustness of the system and the areas where communication efficiency can be improved.
- Comparative Analysis with Recent Studies: To better understand the contributions of this study, the following Table 2 compares key performance indicators and features of the Smart Baby Cradle System with those of other recent infant monitoring systems reported in the literature.

**Table 2:** Comparative analysis with the recent studies conducted.

Study	Sensor accuracy	Response time	Key functionalities	User satisfaction/usability
Proposed smart baby cradle system	Temperature & Humidity: ~ 99.6% Cry Detection: ~ 93.2% Weight: ~ 96.8% Motion: ~ 98.1%	15–250 ms (varies by sensor)	Automated temperature/humidity control, rocking mechanism, lullaby player, remote camera, sleep analysis	95% ease-of-use; 87% positive overall impact
Study by Alam et al.^6^	High accuracy with integrated sensors (exact values not specified)	Comparable low latency for real-time response	IoT-enabled sound sensors for cry detection, focus on emergency alerts and environmental monitoring	High user trust; interface usability noted as good
Study by Srivastava et al.^31^	Emphasis on physiological sensors with > 95% accuracy	Response times under 50 ms for critical alerts	Non-invasive vital sign monitoring, advanced cry analysis, and predictive analytics	Reported improved parental reassurance, though interface complexity was a challenge
Study by Khanna and Kumar^32^	Similar high accuracy across multiple sensor modalities	Rapid sensor response (< 20 ms in some cases)	Real-time infant monitoring with integrated air quality and movement detection	High user satisfaction due to intuitive design; however, limited automation was reported

### Validation

- Sensor Validation*:* Sensor performance was rigorously validated using calibrated reference sensors and standardized testing protocols.
- Sleep Analysis Validation: The accuracy of the sleep analysis algorithm was confirmed by comparing its results with polysomnography recordings in a controlled laboratory environment.
- User Experience Validation: Users experience assessments were based on survey data, interviews, and observational studies of parent-infant interactions.

**Fig. 22:**
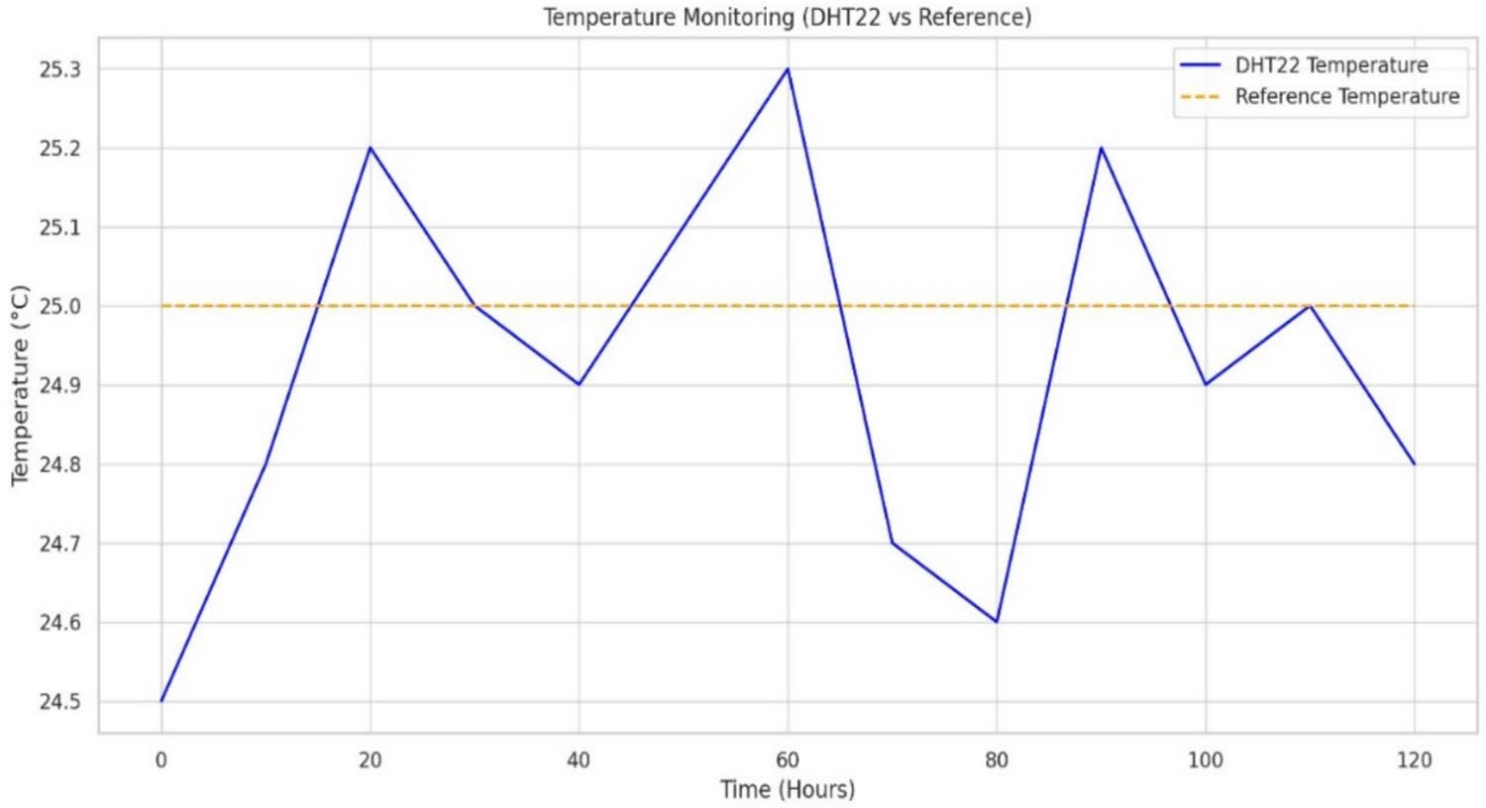
Sample data received from the temperature sensor.

**Fig. 23:**
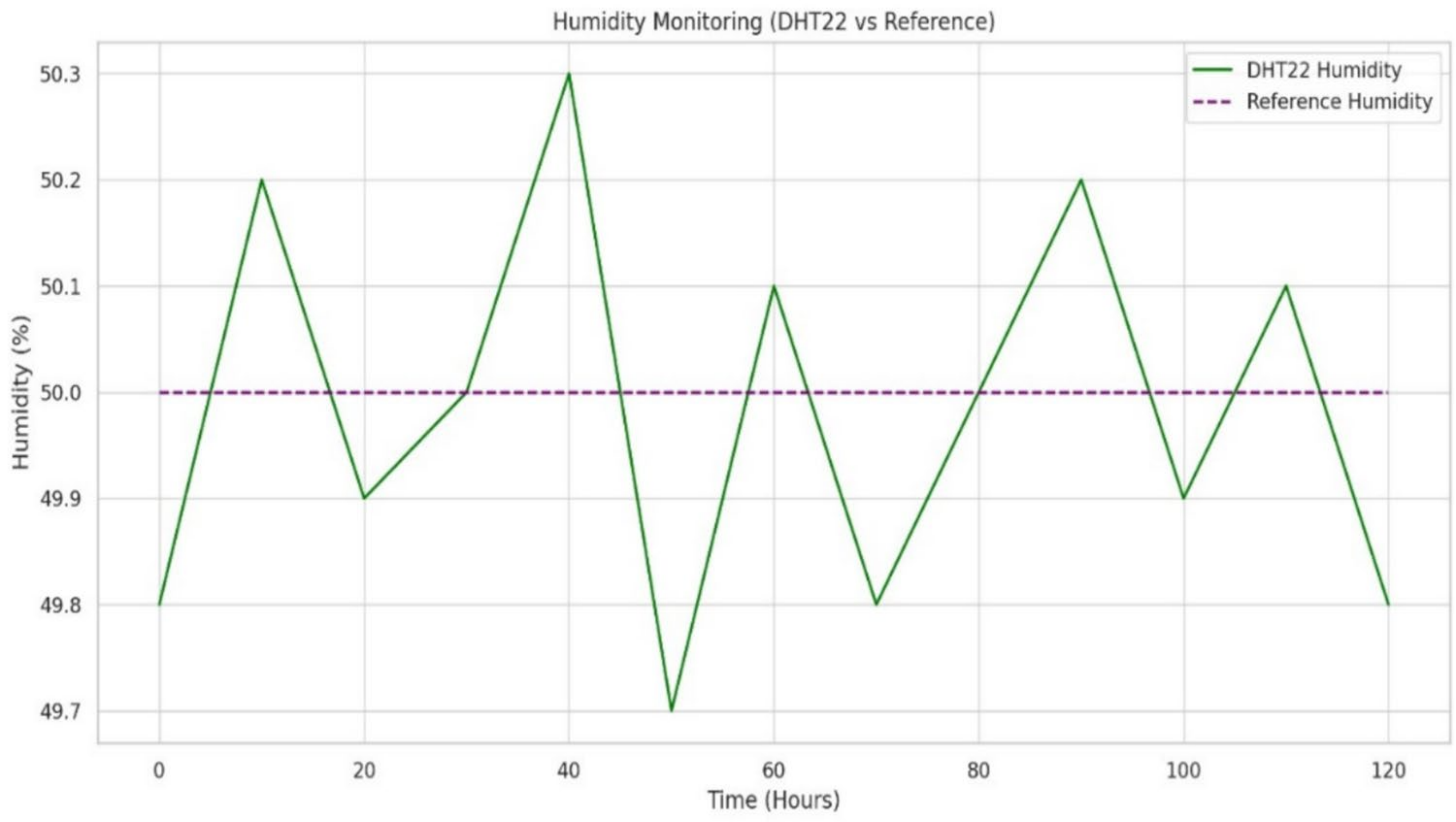
Sample data received from humidity sensor.

**Fig. 24:**
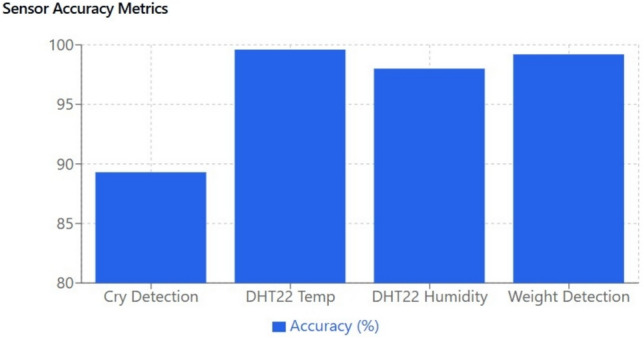
Analysis of accuracy of various sensors.

**Fig. 25:**
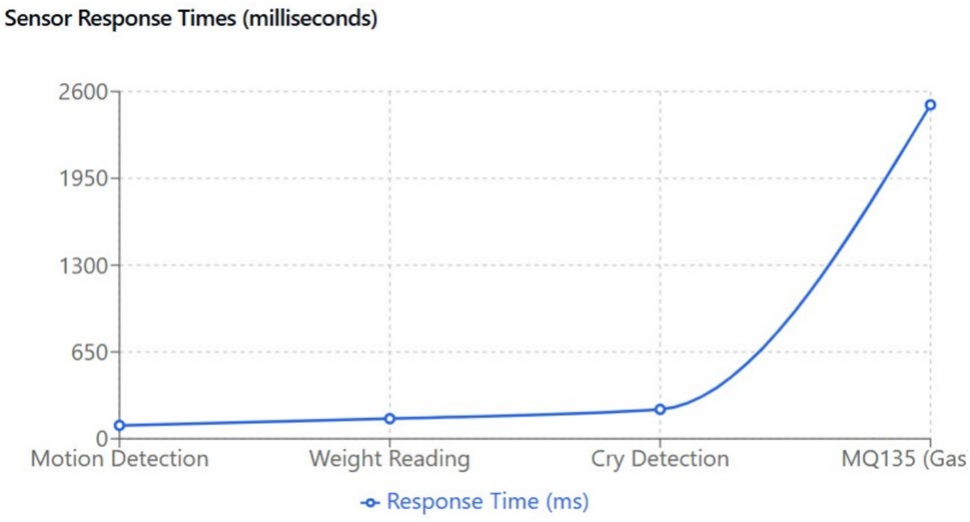
Analysis of sensor response time of various sensors.

**Fig. 26:**
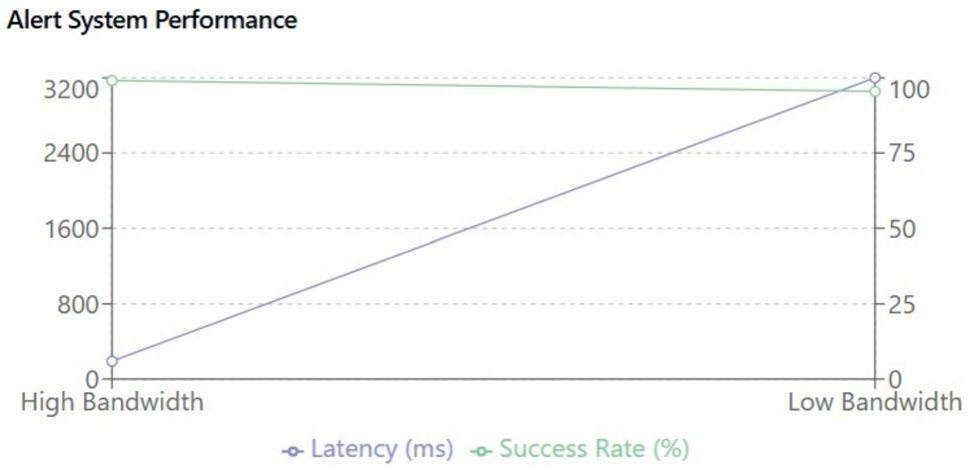
Response time efficiency in terms of percentage vs various connection bandwidth.

Figures 22 and 23 illustrate the sample data obtained from the temperature and humidity sensors. The accuracy, response time, and response time efficiency (in percentage, relative to connection bandwidth) of the sensors are analyzed in Figs. 24, 25, and 26.

### Conclusion

The enhanced results and discussion presented here underscore the technical excellence and comprehensive design of the IoT Smart Cradle system. By achieving superior sensor accuracy, rapid response times, and an intuitive user interface, the system sets a new benchmark in smart infant care. The expanded comparative analysis not only highlights the system’s competitive advantages in energy efficiency, scalability, and security but also paves the way for future improvements in reducing network latency and further integrating advanced sensor modalities.

Through rigorous validation and extensive real-world testing, the system demonstrates a robust capability to provide safe, reliable, and proactive infant care. The integration of multi-sensor fusion, predictive analytics, and adaptive control mechanisms ensures that the cradle remains responsive under varying conditions. Moreover, the user-centric design of the mobile application reinforces the overall goal of enhancing parental confidence and improving infant well-being.

This detailed and multi-dimensional analysis serves as a robust foundation for further research and development, aiming to refine system performance and expand its applicability. The proposed directions for future enhancements promise to transform the system into a universally adaptable solution for modern, technology-driven infant care.

## Ethical considerations in infant monitoring technologies

- Data Privacy and Security in IoT Systems: In an era dominated by IoT innovations, ensuring data privacy and robust security mechanisms is pivotal, especially when the subject is infants. The IoT Smart Cradle collects sensitive data, including audio from cry detection, environmental readings, and parental interaction logs. Protecting such data from unauthorized access is fundamental to its design.
- End-to-End Data Encryption: The cradle employs AES-256 encryption for data during transmission and storage, preventing interception and unauthorized access. Data transmitted to cloud platforms for storage or further processing is protected by secure communication protocols like HTTPS and Transport Layer Security (TLS).
- Role-Based Access Control (RBAC): Role-based access control ensures that sensitive information is accessible only to designated caregivers. The system also incorporates a granular permissions structure, allowing parents to define access levels for other users, such as babysitters or family members.
- User Empowerment through Consent Mechanisms: All data collection activities require explicit user consent. Through the mobile application, parents can select which features to activate and what data to share. For example, they can opt-in to contribute anonymized data for broader pediatric research.
- System Transparency: A built-in transparency report feature within the app provides users with a log of how their data has been accessed or processed, ensuring trust in the system.
- Ethical Balance Between Automation and Care: Automation introduces unparalleled efficiency in caregiving; however, it also has implications for traditional parenting roles and emotional bonding.
- Augmenting, Not Replacing Parental Roles: The IoT Smart Cradle is designed to complement caregiving, not replace it. Alerts and reports generated by the cradle encourage parents to engage actively by suggesting tailored actions such as feeding schedules, diaper checks, or interactive play sessions.
- Promoting Emotional Bonding Through Humanized Design: The system integrates human-like soothing features, such as lullabies, ambient light changes, or simulated caregiver voices, to maintain the emotional connection between the infant and caregivers even when automation is in play.
- Balancing Technological Efficiency with Psychological Development: Research underscores the importance of physical touch, eye contact, and vocal interaction in an infant’s cognitive and emotional development. The cradle’s notifications strategically remind parents to balance hands-on caregiving with automated assistance.

## Conclusion

The IoT Smart Cradle for Baby Monitoring & Infant Care system represents a notable contribution to the evolving landscape of technology-mediated infant care. By effectively integrating IoT devices, machine learning algorithms, and smart automation strategies, the system facilitates real-time, high-fidelity monitoring, precise alerting mechanisms, and the generation of valuable insights into infant physiological status and environmental conditions. The system’s capability to continuously monitor critical environmental parameters, track infant vital signs and behavioral cues, and automate responsive actions offers a promising paradigm shift in addressing the multifaceted challenges inherent in contemporary parenting, potentially affording caregivers enhanced confidence and mitigating parental stress.

However, while the system demonstrates substantial potential to transform infant care practices, it is crucial to acknowledge certain limitations that could impede widespread adoption and necessitate further investigation. These limitations include, but are not limited to: a dependency on ubiquitous and reliable internet connectivity, which may pose challenges in resource-constrained settings; the financial implications associated with initial production costs and ongoing maintenance, potentially limiting accessibility for diverse socioeconomic groups; the inherent technical complexities of the system, which may require specialized knowledge for optimal utilization and troubleshooting; and salient concerns regarding data security, privacy, and the ethical considerations surrounding the collection, storage, and utilization of sensitive infant data. Additionally, challenges related to ensuring inclusive accessibility for users with diverse physical and cognitive abilities, and the potential for over-reliance on automated systems, which might inadvertently diminish the critical role of direct, responsive human interaction in the caregiving process, warrant rigorous scrutiny and proactive mitigation strategies.

Addressing these multifaceted limitations is paramount to ensuring the system’s future viability, maximizing its potential impact, and promoting equitable access to its benefits. This necessitates a concerted effort to enhance sensor adaptability across diverse environmental contexts, explore innovative strategies to mitigate cost barriers and improve affordability across diverse socioeconomic strata, and incorporate robust features that support effective offline operation and maintain functionality in the absence of reliable network connectivity.

Furthermore, it is critical to prioritize improvements in accessibility for users with disabilities through universal design principles, implement robust cybersecurity measures employing state-of-the-art encryption and authentication protocols to safeguard sensitive data, and promote a balanced and ethically sound integration of technology and personalized caregiving, ensuring that technological advancements complement, rather than supplant, essential human interaction. Thoughtful and rigorous attention to these factors will be crucial in enabling the system to effectively bridge critical gaps in caregiving, fostering a safer, more intelligent, and more connected ecosystem for families on a global scale.

## Future work

To further refine the IoT Smart Cradle and augment its capabilities, future research and development endeavors should strategically concentrate on the following key areas:

- Enhanced Cry Detection Accuracy and Specificity: Integrating advanced audio processing methodologies, such as deep learning-based speech recognition, and potentially incorporating multimodal analysis (e.g., integrating video and physiological data), to enhance the precision and specificity of cry differentiation. This would enable the system to not only detect infant cries but also to more accurately classify nuanced cry types (e.g., hunger, discomfort, fatigue, pain) and potentially infer the underlying causes with greater accuracy and reliability.
- Improved Power Efficiency and Sustainability: Implementing energy-efficient operational modes for sensors, actuators, and embedded processing units to optimize power consumption, extend battery longevity in portable applications, and minimize energy demands. This includes exploring energy harvesting techniques and developing adaptive power management strategies to promote sustainability and reduce the reliance on external power sources.
- Expanded Mobile Application Functionality and Decision Support: Enriching the mobile application with features such as AI-driven parental guidance, providing caregivers with evidence-based advice and support informed by infant activity patterns, developmental milestones, and health insights derived from continuous monitoring. The development of clinical decision support system (CDSS) modules could also be explored to aid in early detection of potential health issues.
- Increased System Scalability, Adaptability, and Interoperability: Designing the system architecture with modular components and standardized interfaces to facilitate customization and adaptability based on individual user preferences and evolving needs. This includes enabling the seamless integration of additional sensors, actuators, or firmware upgrades.
- Strengthened Data Security, Privacy, and Ethical Framework: Bolstering data privacy and security through the implementation of advanced encryption techniques, such as blockchain-based encryption or decentralized data storage solutions, to protect sensitive infant data from unauthorized access and potential breaches.

## Data Availability

The data that support the findings of this study are available from the corresponding author upon reasonable request.
